# Divergent Transcriptional Regulatory Logic at the Intersection of Tissue Growth and Developmental Patterning

**DOI:** 10.1371/journal.pgen.1003753

**Published:** 2013-09-05

**Authors:** Matthew Slattery, Roumen Voutev, Lijia Ma, Nicolas Nègre, Kevin P. White, Richard S. Mann

**Affiliations:** 1 Department of Biochemistry and Molecular Biophysics, Columbia University, New York, New York, United States of America; 2 Institute for Genomics and Systems Biology and Department of Human Genetics, University of Chicago, Chicago, Illinois, United States of America; 3 Université de Montpellier 2 and INRA, UMR1333 DGIMI, Montpellier, France; New York University, United States of America

## Abstract

The Yorkie/Yap transcriptional coactivator is a well-known regulator of cellular proliferation in both invertebrates and mammals. As a coactivator, Yorkie (Yki) lacks a DNA binding domain and must partner with sequence-specific DNA binding proteins in the nucleus to regulate gene expression; in *Drosophila*, the developmental regulators Scalloped (Sd) and Homothorax (Hth) are two such partners. To determine the range of target genes regulated by these three transcription factors, we performed genome-wide chromatin immunoprecipitation experiments for each factor in both the wing and eye-antenna imaginal discs. Strong, tissue-specific binding patterns are observed for Sd and Hth, while Yki binding is remarkably similar across both tissues. Binding events common to the eye and wing are also present for Sd and Hth; these are associated with genes regulating cell proliferation and “housekeeping” functions, and account for the majority of Yki binding. In contrast, tissue-specific binding events for Sd and Hth significantly overlap enhancers that are active in the given tissue, are enriched in Sd and Hth DNA binding sites, respectively, and are associated with genes that are consistent with each factor's previously established tissue-specific functions. Tissue-specific binding events are also significantly associated with Polycomb targeted chromatin domains. To provide mechanistic insights into tissue-specific regulation, we identify and characterize eye and wing enhancers of the Yki-targeted *bantam* microRNA gene and demonstrate that they are dependent on direct binding by Hth and Sd, respectively. Overall these results suggest that both Sd and Hth use distinct strategies – one shared between tissues and associated with Yki, the other tissue-specific, generally Yki-independent and associated with developmental patterning – to regulate distinct gene sets during development.

## Author Summary

The Hippo tumor suppressor pathway controls proliferation in a tissue-nonspecific fashion in *Drosophila* epithelial progenitor tissues via the transcriptional coactivator Yorkie (Yki). However, despite the tissue-nonspecific role that Yki plays in tissue growth, the transcription factors that recruit Yki to DNA, most notably Scalloped (Sd) and Homothorax (Hth), are important regulators of developmental patterning with many tissue-specific functions. Thus, these three transcriptional regulators – Yki, Sd, and Hth – provide a model for exploring the properties of protein-DNA interactions that regulate both tissue-shared and tissue-specific functions. With this goal in mind, we identified the positions in the fly genome that are bound by Yki, Sd, and Hth in the progenitors of the wing and eye-antenna structures of the fly. These data not only provide a global view of the Yki gene regulatory network, they reveal an unusual amount of tissue specificity in the genomic regions targeted by Sd and Hth, but not Yki. The data also reveal that tissue-specific binding is very likely to overlap tissue-specific enhancer regions, provide important clues for how tissue-specific Sd and Hth binding occurs, and support the idea that gene regulatory networks are plastic, with spatial differences in binding significantly impacting network structures.

## Introduction

The regulation of gene expression is a complex, multilayered process, but at its core lays the interaction between transcription factors (TFs) and DNA. TFs regulate gene expression by binding their target DNA sequences, which are generally organized into groups of regulatory motifs known as enhancers or *cis*-regulatory modules (CRMs) [1]–[3]. Understanding how TFs interact with DNA is crucial for our understanding of gene regulatory networks, and genomic approaches – chromatin immunoprecipitation followed by microarray or sequencing analysis (ChIP-chip or ChIP-seq, respectively) – have now given us the ability to monitor TF-DNA interactions on a genome-wide scale [4]–[6]. However, understanding the regulatory impact of the observed interactions remains a challenge, especially in light of the fact that many TFs appear to bind to thousands of genomic regions [7]–[9]. Thus one of the key questions now faced by those attempting to map regulatory networks is how regulatory specificity is achieved within this sea of TF-DNA binding.

It is likely that only a subset of the thousands of binding events observed for most TFs regulate gene expression. Work on the *Drosophila* early embryo TF network suggests that functional binding can be distinguished from neutral binding based simply on ChIP signal strength, and studies exploring the fly embryonic mesoderm TF network indicate that temporally dynamic binding is more likely to be functional [10]–[13]. While the former study is based on a single developmental time point (the blastoderm stage of embryogenesis), the latter studies suggests developmentally dynamic TF-DNA interactions play a crucial role in defining the gene regulatory networks at later stages of development. Furthermore, additional studies have highlighted the importance of tissue and chromatin context in impacting TF-DNA interactions in *Drosophila* [14]–[18] and mammals [19]–[21]. Clustered binding events – possibly representing ‘shadow’ or ‘distributed’ enhancers – have also been highlighted as enriched in functional binding [13], [22]–[25] and, accordingly, regions of clustered ChIP peaks are more likely to be developmentally dynamic [25]. Indeed, the regulatory networks of later developmental stages may be more complex than those of the early embryo. As the development of multicellular organisms proceeds, cell fates are progressively refined, generating numerous cell and tissue types throughout the organism; growth and patterning of these unique tissues often requires the reiterative use of a largely overlapping set of TFs [26]. If the same TFs are reused in different tissue types to carry out distinct functions, precise mechanisms must be in place for these factors to achieve regulatory specificity.

One possible scenario for tissue-specific TF functions is that the same TF binds to distinct DNA sequences in a tissue-specific fashion. In this model, tissue-specific CRM activities are directed by tissue-specific TF-DNA interactions. Conversely, tissue-shared TF-DNA interactions would drive tissue-nonspecific CRM activity across tissues. Tissue-specific binding could be regulated through direct or indirect interactions with other transcription factors, or through tissue-specific differences in chromatin landscape, such as binding site accessibility or histone modifications [1], [2], [27]–[30]. In an alternate model, the tissue-specific regulatory activity of a TF is regulated at a step subsequent to DNA binding [26]. In this case, binding events shared between tissues can drive tissue-specific expression patterns, with regulatory specificity provided by direct or indirect interactions with another transcription factor or cofactor. Although tissue-specific binding is thought to reveal functional enhancers [2], [11], [12], [31], it remains an open question whether tissue-nonspecific binding of TFs is functional and, if so, whether it can also lead to tissue-specific enhancer activity.

Regardless of whether a TF's activity is regulated at the level of DNA binding or beyond, chromatin landscape has the potential to modulate regulatory output. The histones that make up nucleosomes can be subject to significant posttranslational modification, and certain posttranslational modifications are associated with active or inactive CRMs (e.g. histone 3 lysine 27 acetylation or trimethylation, respectively) [32], [33]. A recent genome-wide study of >50 chromatin-associated proteins found that *Drosophila* chromatin can be broken down into five distinct chromatin states: YELLOW, RED, BLUE, BLACK, and GREEN [34], [35]. The YELLOW and RED states represent generally ‘active’ chromatin, while the other three represent various ‘repressive’ states. This five state model is based on the DamID (DNA adenine methyltransferase identification) method for characterizing *in vivo* protein-DNA interactions, but is highly consistent with a similar model based on genome-wide ChIP data [34], [36], [37]. Although much is yet to be explored regarding the interplay of TFs and these chromatin types, the five DNA-binding factors tested in the chromatin state study preferentially bound RED chromatin, suggesting this chromatin state might positively modulate DNA interactions for these factors [34]. However, as these studies were conducted in cell lines, the influence of chromatin type on tissue-specific binding and regulatory activity *in vivo* remains untested.

To begin exploring the mechanisms underlying tissue-specific gene regulation, we focus here on three *Drosophila* transcriptional regulators that have been implicated downstream of the Hippo signaling pathway: Yorkie (Yki), Scalloped (Sd), and Homothorax (Hth). The Hippo tumor suppressor pathway is a key regulator of cellular proliferation in both invertebrates and mammals [38]–[42]. The pathway centers around two serine-threonine kinases, Hippo and Warts, and downstream of these kinases the Hippo pathway regulates gene transcription [43], [44]. A direct target of Warts, the transcriptional coactivator Yki is an essential mediator of Hippo-regulated proliferation [45]. As a coactivator, Yki lacks a DNA binding domain and must partner with sequence-specific DNA binding proteins in the nucleus to regulate gene expression. Multiple TFs have been implicated in the recruitment of Yki to DNA; in *Drosophila*, two well-characterized Yki binding partners are Sd and Hth [46]–[51]. Yki promotes tissue growth in a tissue-nonspecific manner across imaginal discs, and Sd and Hth are necessary for these functions in the wing and eye, respectively [48]–[50]. Ectopic Yki activity, whether driven by targeted overexpression or through mutations that compromise Hippo signaling, drives tissue overgrowth without changing tissue identity [45]. Sd and Hth, on the other hand, are required for both tissue identity and tissue growth: in addition to their roles regulating proliferation together with Yki, Sd and Hth also have important Yki-independent developmental roles. For example, Sd, in conjunction with Vestigial (Vg), specifies wing fate [52]–[54]. Hth specifies antennal fate, participates in patterning the proximal-distal axis of the wing and leg, and maintains cells in an undifferentiated state in the developing eye [55]–[59]. Additionally, Yki and Sd play a role in specifying non-retinal fates in the eye imaginal disc [60]. Thus, these three factors are ideal for studying context-specific gene regulation: all three factors promote tissue growth (cell proliferation and survival), while Sd and Hth also carry out highly tissue-specific functions.

Because of their unique and shared roles in the wing and eye-antennal imaginal discs, we performed genome-wide ChIP experiments for Sd, Hth, and Yki in both of these tissues. Strong, tissue-specific binding patterns are observed for Sd and Hth, while Yki binding is remarkably similar between these two tissues. Tissue-specific binding events for Sd and Hth are located at genes consistent with their known developmental roles, are significantly enriched in Polycomb-associated (BLUE) chromatin, and are associated with enhancers that are active in the corresponding tissue. Binding events common to the eye and wing are also observed for Sd and Hth; these tissue-shared binding events are generally associated with genes regulating cell proliferation and other “housekeeping” functions. Interestingly, the tissue-shared Hth and Sd binding events account for the majority of Yki occupancy. We also identified and characterized separate but adjacent wing and eye enhancers from the *bantam* (*ban*) gene, a previously described direct target of the Hippo pathway, and show that their activities are dependent on direct Sd and Hth binding, respectively. Overall these results suggest that the TFs Sd and Hth use at least two binding strategies – one context-independent and associated with Yki binding, the other tissue-specific and associated with developmental patterning – to regulate different gene sets during development.

## Results

### Overview of Yki, Sd, and Hth binding events

**Figure 1:**
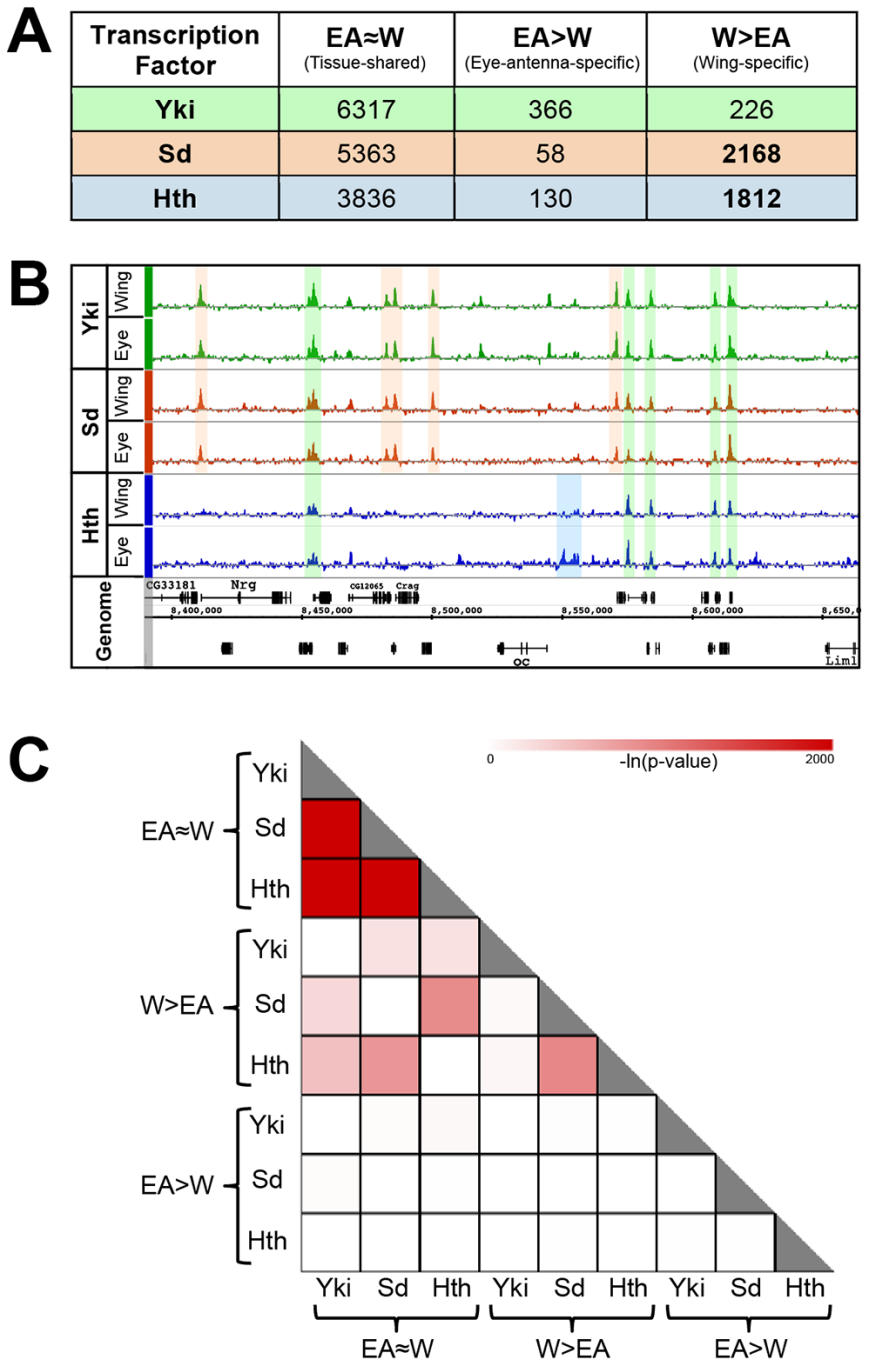
Overview of genome-wide Yki, Sd, and Hth binding patterns. (A) Summary of tissue-shared (EA≈W) and tissue-specific (W>EA or EA>W) binding events for Yki, Sd, and Hth. Peaks called at FDR1 in one tissue and not called at FDR25 in the opposite tissue are considered tissue-specific. Peaks called at FDR1 in one tissue and ≤FDR25 in the opposite tissue are considered tissue-shared. (B) Yki, Sd, and Hth binding profiles in the eye-antenna and wing imaginal discs across approximately 250 kb of Chromosome X. Various combinations of binding overlap and tissue-specificity are highlighted by the colored shading. (C) Heatmap representing the significance of overlap in binding between tissue-shared (EA≈W) and tissue-specific (W>EA or EA>W) categories of Yki, Sd, and Hth binding. Shading represents −ln(p-value) for each pair-wise comparison.

The transcriptional coactivator Yki is required for cell survival in all imaginal discs [45]. Two of Yki's partner TFs, Hth and Sd, are required for cell survival in the eye and wing imaginal discs, respectively, yet these TFs also have important developmental roles beyond the control of cell proliferation and survival. To explore tissue specific gene regulation by these TFs at the downstream end of the Hippo pathway, we performed genome-wide chromatin immunoprecipitation (ChIP-chip) experiments for each factor in both the wing (W) and eye-antenna (EA) imaginal discs. For Hth and Yki we used polyclonal antibodies raised against the native proteins, and for Sd we used a GFP protein trap line, which is wild type as a hemi- or homozygote, and polyclonal anti-GFP to immunoprecipitate bound chromatin fragments from wild type eye-antenna or wing imaginal discs of wandering stage 3^rd^ instar larvae [61]–[63]. Immunoprecipitated fragments were hybridized to high-density, whole-genome tiling arrays to generate a global, tissue-specific view of genomic binding for all three factors (Figure 1A,B).

An overview of the binding events for these factors is provided in Figure 1A and extensive lists are provided in Dataset S1. To explore the tissue specificity of Yki, Sd and Hth binding, we defined tissue-specific peaks as those that are called at a False Discovery Rate (FDR) of 1% in the tissue of interest and not called at a less stringent FDR of 25% in the other tissue (Figure 1A) [64]. This dual-threshold method avoids calling a peak as tissue-specific if it falls just below the significance threshold of FDR1 in one of the two tissues (i.e., a peak that would be called at FDR1 in the eye and an FDR of 5% in the wing). Although small differences in binding strength may also be important for tissue specific gene regulation, our initial goal was to characterize robust tissue specific binding events. For simplicity, W>EA will be used to refer to bound regions called as FDR1 peaks in the wing and not called as FDR25 peaks in the eye-antenna; the converse will be referred to as EA>W. Regions called as FDR1 peaks in one tissue and at FDR25 in the other tissue are considered as shared binding events in both tissues, and referred to as EA≈W binding events. Using this thresholding scheme (Figure 1A), it is immediately apparent that Sd and Hth specifically bind a large number of genomic regions in the wing disc (approximately 2000 W>EA for both factors) compared to the eye-antennal disc (<200 EA>W for both factors). In contrast, for both tissues, tissue-specific binding by Yki is limited to a few hundred events, a small fraction of the total (less than 6%). Thus, the tissue-specific binding events observed for both Sd and Hth distinguish these factors from Yki, which displays little tissue-specific binding (Figure 1A). These results suggest that the site specific TFs Sd and Hth target the genome in a way that is fundamentally distinct from the coactivator Yki; in these two imaginal tissues, Sd and Hth binding is exquisitely sensitive to cellular context, whereas Yki binding is relatively insensitive to cellular context.

As Yki lacks a DNA binding domain, DNA binding TFs such as Sd and Hth are needed for recruitment of Yki to regulatory loci. To determine the extent to which Sd and Hth can account for Yki binding we compared the genome-wide binding site overlap between Yki and these two TFs. In total, Sd and Hth can account for ∼70% of Yki binding in wing, and ∼50% of Yki binding in the eye-antenna (Figure S1, see also Figure 1C discussion below). Because of the difficulties inherent in comparing independently thresholded binding site calls, this is likely to be a conservative estimate. Indeed, if we instead ask how many Yki binding sites overlap Sd and Hth peaks called at an FDR of 25%, we find that these two factors overlap 82% in the wing and 73% in the eye (not shown). Regardless of the peak-calling threshold used, Yki's overlap with Sd is more prevalent than its overlap with Hth, suggesting Sd is used more frequently than Hth to recruit Yki in both tissues (Figure S1).

Consistent with the finding that the majority of Yki binding is shared between the wing and eye-antenna discs, Yki's EA≈W peaks overlap most significantly with EA≈W peaks for Sd and Hth (Figure 1C). In fact, the EA≈W binding events for all three factors are highly correlated. For Yki over 25% of EA≈W peaks overlap both Sd and Hth EA≈W peaks (all three factors bound to the same location), and two-thirds overlap with at least one of the two TFs. The pattern is more dramatic for Sd and Hth. Approximately 37% of Sd EA≈W peaks overlap Yki+Hth peaks and ∼88% overlap Yki or Hth peaks. For Hth, almost half (49%) of the EA≈W peaks overlap Yki+Sd peaks and 72% overlap Yki or Sd. The high overlap of tissue-shared binding is reminiscent of previously described ‘hotspots’ of TF colocalization, or HOT (high-occupancy target) regions [37], [65], [66]. Indeed, the EA≈W binding events for all three factors significantly overlap embryonic HOT regions, with 57%, 53%, and 37% of HOT regions overlapping EA≈W Yki, Sd, and Hth, respectively (all p<10^−50^, hypergeometric test). Thus, the bound regions shared by these three factors in the imaginal discs are also significantly bound by other TFs at a very different stage of development. On the other hand, overlap between Yki binding with tissue-specific Sd and Hth binding events is not nearly as significant (Figure 1C). These results indicate that when Yki, Sd, and Hth are bound to the same genomic locations, this co-occupancy is independent of tissue context.

### Tissue-specific versus tissue-shared binding

It is clear from the results described above that two distinct types of binding are observed for the TFs Sd and Hth: binding that is shared between the wing and the eye-antenna, and binding that is specific to one of the two tissues. To better understand the variables influencing tissue-specific binding and, presumably, regulatory specificity, we explored the differences between these two modes of genomic binding.

**Figure 2:**
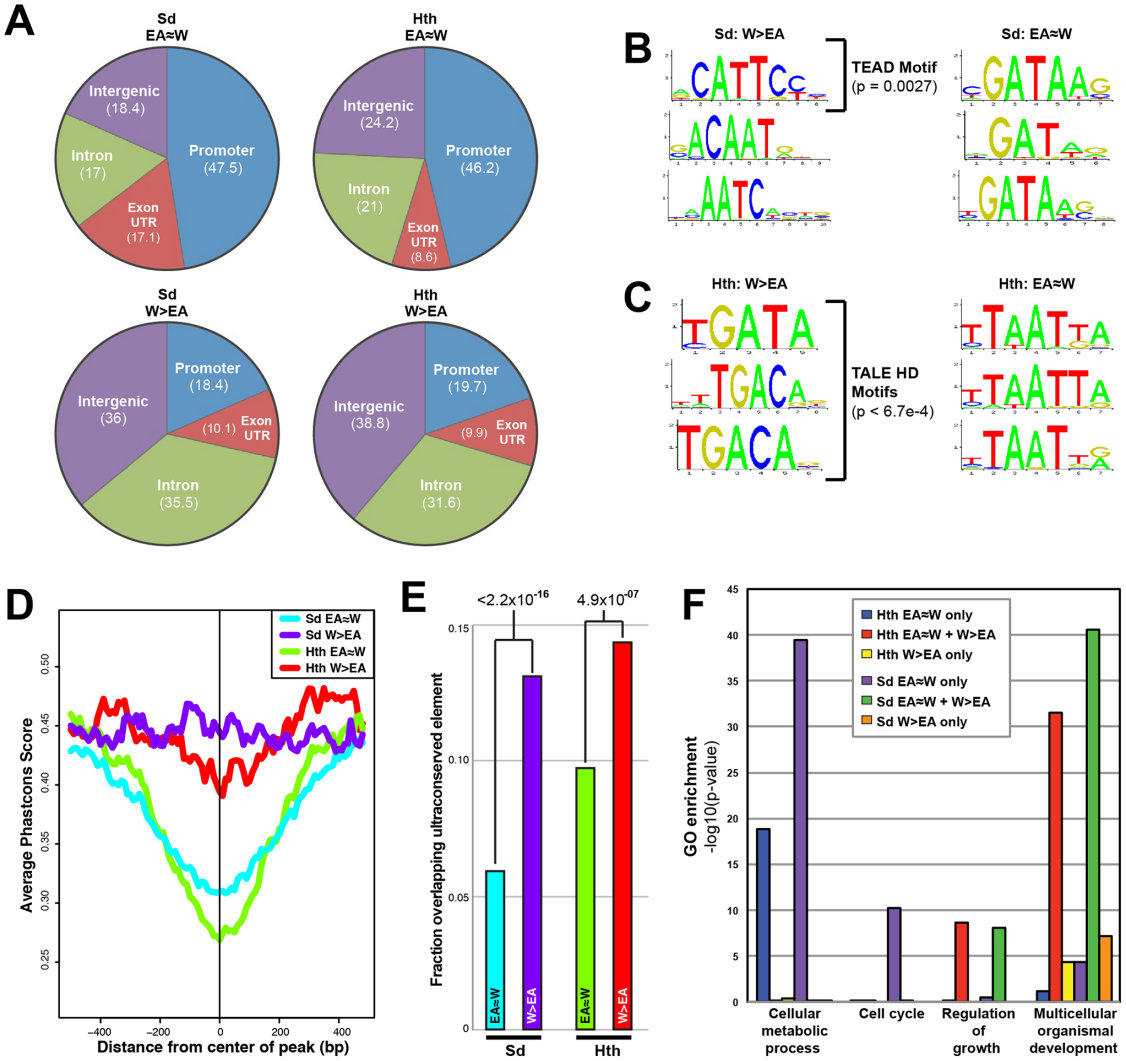
Comparison of tissue-shared and tissue-specific Sd and Hth binding. (A) Pie charts comparing the percentage binding near promoters (within 1 kb upstream of a transcription start site), intron, exon (exon+UTRs), and intergenic regions for EA≈W and W>EA Sd and Hth binding peaks. (B) Top 3 enriched motifs as identified by Centrimo [99] for EA≈W and W>EA Sd binding regions. Enrichment p-values are indicated for the motif matching the Sd consensus (TEAD motif). (C) Top 3 enriched motifs as identified by Centrimo [99] for EA≈W and W>EA Hth binding regions. All three W>EA motifs have p-values <6.7×10^−4^; the first motif matches Exd's consensus and the other two motifs match the Hth consensus sequence. (D) Multispecies conservation score (PhastCons) for EA≈W Sd (blue), EA≈W Hth (green), W>EA Sd (purple), and W>EA Hth (red) binding regions. Average score for a 1000 bp window (center of called peak +/−500 bp) is represented. (E) Fraction of EA≈W Sd (blue), EA≈W Hth (green), W>EA Sd (purple), and W>EA Hth (red) peaks that overlap an ultraconserved element as defined in [109]. Ultraconserved elements are defined as regions of at least 50 bp that are perfectly conserved between *Drosophila melanogaster* and *Drosophila pseudoobscura*. (F) Gene Ontology (GO) enrichment across gene sets targeted by a Sd and Hth tissue-shared binding only (‘EA≈W only’), tissue-shared and tissue-specific binding (‘EA≈W+W>EA’), or tissue-specific binding only (‘W>EA only’) event.

We first sought to further characterize the DNA targeted by tissue-specific and tissue-shared Sd and Hth binding. EA>W binding events were left out of these analyses because it is difficult to compare patterns from the small number of binding events in this set to patterns from the thousands of binding events in the tissue-shared and W>EA sets. We looked at three additional characteristics – genomic location (TSS proximal, intergenic, intronic, etc.), DNA motif enrichment, and DNA conservation – and, again, found striking differences between the tissue-shared and W>EA binding sites. First, tissue-shared binding is much more likely to fall at proximal promoter regions, with >46% of EA≈W for both Sd and Hth falling within 1 kb of a transcription start site. In contrast, W>EA binding is much more likely to occur in intronic or intergenic regions, with >70% of W>EA binding for both Sd and Hth falling within intergenic or intronic DNA (Figure 2A).

With regard to potential DNA motifs influencing Sd and Hth binding, the most significant centrally enriched motifs in EA≈W peaks do not match characterized Sd or Hth DNA binding sites, but instead are GATA-like motifs and AT-rich motifs, respectively (Figure 2B,C). Interestingly, Sd and Hth sequences matching the consensus DNA binding sites are the most enriched in the W>EA binding regions for each factor, respectively (Figure 2B,C) [53], [67]–[71]. A complete list of enriched motifs is provided in Dataset S2 (see also Figure S2). We also find that, for both Sd and Hth, sequences at W>EA binding events are more likely to be evolutionarily conserved compared to sequences at EA≈W binding events (Figure 2D,E). Thus, binding events that are dependent on tissue context are distinct from shared binding events in multiple ways – they are more likely to be distal to the transcription start site, associated with expected DNA motifs, and more conserved. All of these qualities are consistent with tissue-specific binding events occurring at CRMs targeting genes with complex regulatory inputs.

### Hth and Sd regulatory logic

In terms of target genes, for both Sd and Hth EA≈W regions significantly target housekeeping genes, but additional non-housekeeping (i.e., developmental) gene classes are also targeted (Dataset S3), which is not surprising considering the thousands of Sd and Hth EA≈W binding events. RNA-seq data from WT wing discs reveal that the majority of genes with Sd and Hth EA≈W are highly expressed (Dataset S4). We also asked whether tissue-specific and tissue shared events ever target the same loci, or whether these two modes of binding are always separable. For both Sd and Hth, we separated target genes into those targeted only by an EA≈W binding event (termed ‘EA≈W only’), those targeted by both EA≈W and W>EA binding (termed ‘EA≈W+W>EA’), and those targeted by only W>EA binding (termed ‘W>EA only’).

The majority of EA≈W binding for both Sd and Hth (74% and 68%, respectively) falls into the ‘EA≈W only’ category. The genes targeted in this way – no tissue-specific input – are enriched for housekeeping gene ontology categories like ‘cellular metabolic process’ (Figure 2E); Sd ‘EA≈W only’ also targets cell cycle genes (Figure 2E). Consistent with these GO categories, the ‘EA≈W only’ binding events for both TFs are also the most significantly associated with Yki binding (Figure S1C). In contrast, for both Sd and Hth, the ‘EA≈W only’ events do not significantly target developmental genes. The remaining ∼25% of EA≈W binding for Sd and Hth are associated with loci that also receive tissue-specific input. Genes targeted in this fashion are enriched for categories associated with developmental patterning and morphogenesis (Figure 2E). Thus, both housekeeping genes and developmental genes are associated with tissue-shared input, but developmental genes also have tissue-specific input at distinct locations, perhaps reflecting different CRMs. These results highlight the differences in regulatory logic across unique gene sets, and are consistent with patterned developmental gene expression requiring more complex *cis*-regulatory input.

### CRM targeting by Yki, Sd, and Hth

**Figure 3:**
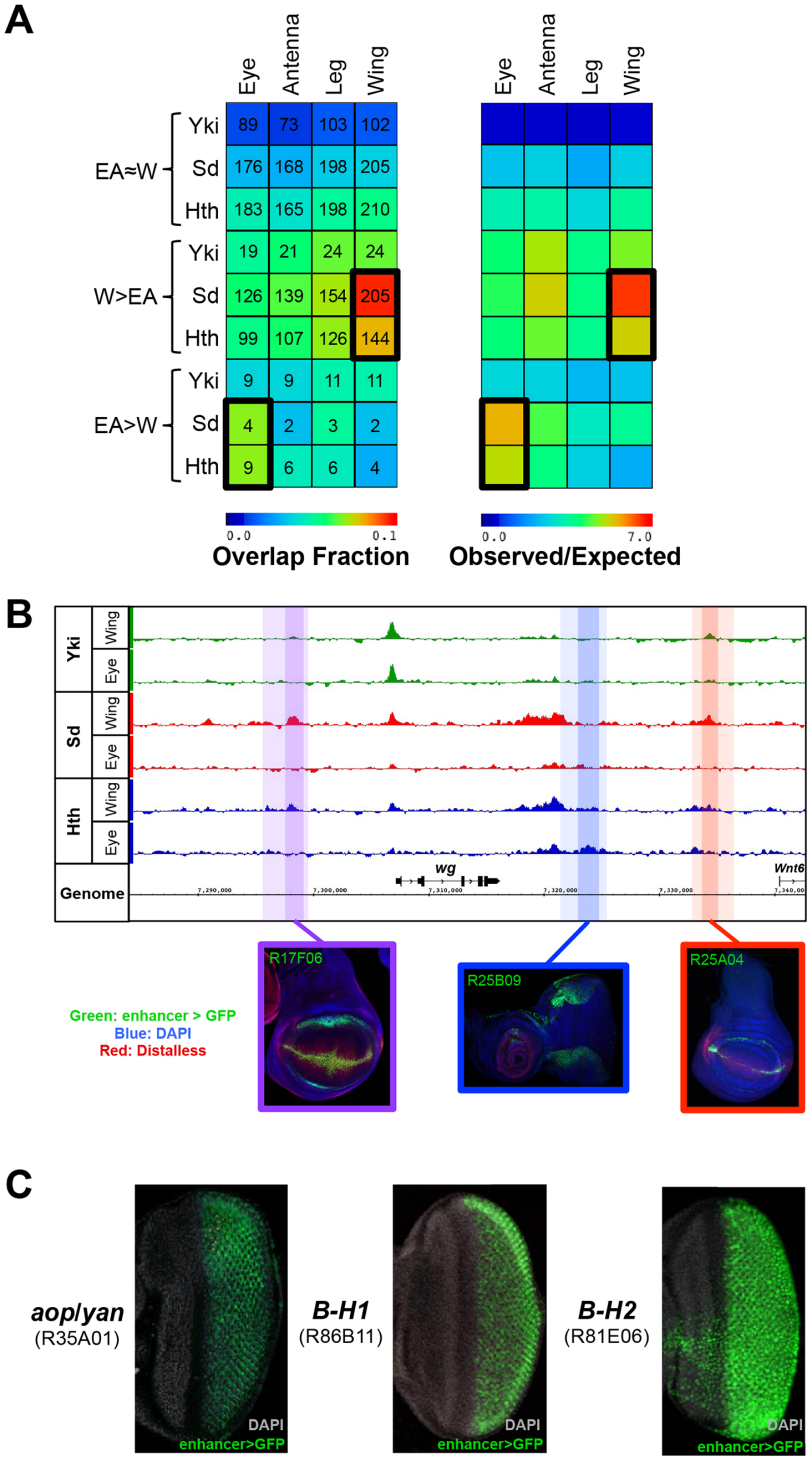
Binding site overlap with imaginal disc enhancers. (A) Left panel: a heatmap representing the fraction of EA≈W, W>EA, and EA>W Yki, Sd, and Hth binding regions that overlap FlyLight enhancers characterized as active in a given tissue (eye, antenna, leg, or wing). Shading represents the fraction of ChIP peaks overlapping an enhancer for each pair-wise comparison, and numbers represent the actual number of binding events overlapping an enhancer. Right panel: a heatmap with the same configuration as the left panel, only shading represents the observed/expected ratio for each pairwise comparison. (B) Yki, Sd, and Hth binding profiles at the *wingless* (*wg*) locus. Lighter shading indicates FlyLight enhancer boundaries – R17F06 is purple, R25B09 is blue, and R25A04 is red – and darker shading indicates binding regions within the enhancers. Expression images for the corresponding enhancers are below the binding profiles; in all three imaginal disc images enhancer-Gal4>UAS-GFP signal in green, DAPI staining in blue, and Distalless staining in red. (C) Expression of putative Sd target enhancers in the eye. From left to right, enhancers near *anterior open* (*aop*, also known as *yan*), and the *Bar* genes *B-H1* and *B-H2* are represented. Images in B) and C) were taken from the FlyLight database [72], [96] and can be found at <http://flweb.janelia.org/cgi-bin/flew.cgi>.

Transcriptional regulators influence gene expression by binding to CRMs, or enhancers. To identify potential CRMs regulated by Yki, Sd, and Hth, we compared our genome-wide binding data to the recently described FlyLight resource cataloging DNA regions with *cis*-regulatory activity in imaginal discs [72]. In total, Sd and Hth each bound >200 DNA fragments that drive expression in the wing disc (248 and 233, respectively), and 170 DNA fragments that drive expression in the eye disc (Dataset S5). Yki binding to FlyLight enhancers was much lower, with Yki peaks overlapping 98 wing enhancers and 84 eye enhancers. In contrast to Sd and Hth, overall Yki binding is not enriched relative to random expectation at FlyLight enhancers. Strikingly, the pattern of TF-CRM colocalization is significantly greater for tissue-specific binding events compared to tissue-shared binding events (Figure 3A). For both Sd and Hth, W>EA binding events are most significantly enriched for enhancers that drive expression in the wing disc, relative to enhancers that drive expression in the eye, antenna, or leg. For example, 164 FlyLight enhancers include Sd W>EA binding peaks and 147 (89.6%) of these are active in the wing. For comparison, only 89 (55%) of the 164 Sd W>EA bound enhancers are active in the eye, and the vast majority of these (80/89, 90%) also drive expression in the wing. A similar pattern is observed for Hth: 131 enhancers have Hth W>EA peaks and 116 (88.6%) of these are active in the wing. On the other hand, 72 (54%) are active in the eye, and 90% (65/72) of these are also active in the wing. Thus, for both Sd and Hth W>EA binding is strongly enriched for enhancers that drive expression in the wing disc. CRMs that are active in the wing but do not overlap with Sd or Hth W>EA binding may be targeted by factors not analyzed here. Although based on a much smaller number of binding events, EA>W binding events are enriched for CRMs driving eye expression (Figure 3A).

In total, W>EA Sd binding events overlap 147 FlyLight DNA fragments that drive expression in the wing (p<10^−95^). Because some of the FlyLight fragments are partially overlapping, this amounts to 115 unique CRMs. For Hth, W>EA binding overlaps 116 enhancer fragments (p<10^−50^), representing 92 unique CRMs. For example, W>EA Sd and Hth binding sites at the *wingless* (*wg*) locus overlap two CRMs that drive expression matching the known *wg* pattern (Figure 3B). Sd is necessary for the dorsal-ventral (DV) stripe of *wg* expression in the wing, and Hth positively regulates *wg* expression in the hinge [54], [59]. Interestingly, one of these *wg* CRMs is bound by both Sd and Hth and captures robust wing DV stripe and hinge expression; only Sd binds the other CRM, which drives DV stripe expression but very weak hinge expression.

The numbers are much smaller, but still significant, in the eye-antenna disc due to the smaller number of EA>W binding events. Hth EA>W binding events overlap 8 CRMs that drive expression in the eye (p<10^−3^), and Sd EA>W binding events overlap 4 eye CRMs (p<10^−2^). Though small in number, interesting patterns are driven by these eye CRMs. Sd, for example, binds 4 CRMs, but all are near genes that play key roles in photoreceptor specification: *anterior open* (*aop*, also known as *yan*), *scabrous* (*sca*), and the *Bar* genes *B-H1* and *B-H2* (Figure 3C) [73], [74]. Hth EA>W binding is associated with CRMs targeting key regulators of eye disc development such as *pointed* (*pnt*), *odd paired* (*opa*), *eyes absent* (*eya*), which is known to be repressed by Hth in the anterior eye, and *wg*, which is positively regulated by Hth in the ventral eye [55], [73], [75]–[77]. Importantly, the *wg* CRM with Hth EA>W binding captures the dorsal and ventral expression domains of *wg* in this tissue (Figure 3B). This CRM is distinct from the wing CRM with Hth W>EA binding (see above) and is consistent with the known role for Hth in the regulation of the *wg* locus [75]. Taken together, these data demonstrate that, in comparison to Yki binding and tissue-shared Sd and Hth binding, tissue-specific binding for both Sd and Hth is more significantly associated with developmentally regulated CRMs often located within intricately regulated loci.

### Hth and Sd regulation of *bantam*

**Figure 4:**
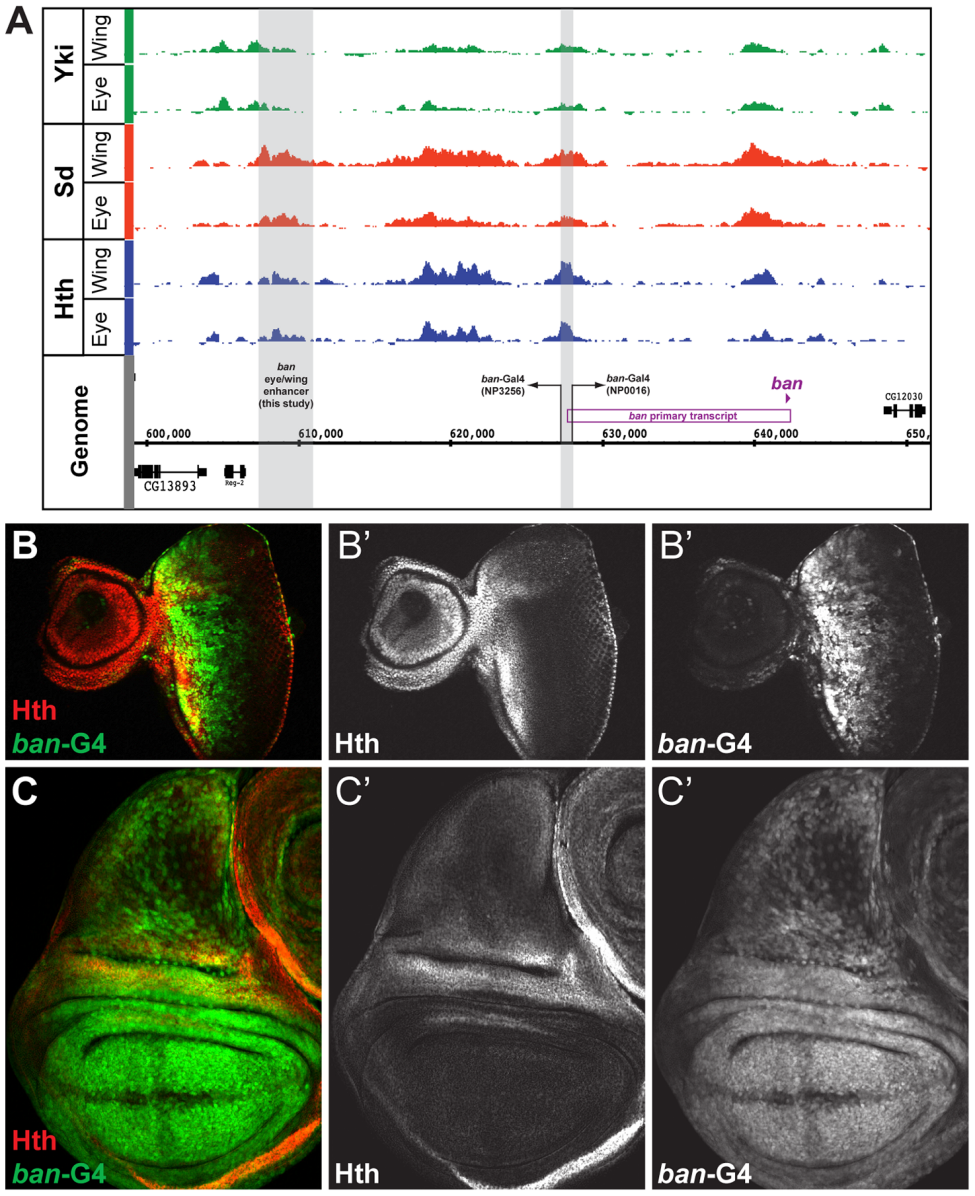
Yki, Sd, and Hth binding at the *bantam* locus. (A) Yki, Sd, and Hth binding profiles in the eye-antenna and wing imaginal discs across the *bantam* locus. The location and directionality of two *ban-Gal4* enhancer traps (NP3256 and NP0016) are indicated by the grey shading, as is the location of the eye/wing enhancer identified in this study. (B and C) *bantam* expression in the (B) eye and (C) wing imaginal discs as captured by the NP0016 enhancer trap; Discs Large (Dlg) staining is red, Hth is blue (also shown in B′ and C′), and *ban-Gal4^NP0016^*>UAS-GFP in green (also shown in B″ and C″). These patterns match those previously revealed by a *ban* sensor transgene [48], [110].

The above findings indicate that tissue-specific binding is a key variable influencing the regulatory specificity of Sd and Hth. Still, a significant fraction of binding for both TFs is tissue-nonspecific, at least when comparing entire eye-antenna and wing imaginal discs; some of the binding that appears to be ‘shared’ could be a consequence of specific binding in distinct cell types within these discs. For example, we observe Sd, Hth, and Yki binding to several well-characterized transcriptional targets of the Hippo pathway in a primarily tissue-nonspecific manner (Figure 4A, Figure S3), including the microRNA (miR) encoding gene *bantam* (*ban*) [48]–[50], [78]–[80]. *ban* both promotes proliferation and prevents apoptosis, and *ban* is essential for Yki-driven overproliferation across imaginal tissues [78]–[80]. Moreover, Sd and Hth regulate *bantam* expression in the wing and the eye, respectively [48]–[50]. Although *ban* expression is patterned in both the wing and eye, the regulatory enhancers that direct these expression patterns have not been previously identified.

Based on transcriptome data and position of putative insulator elements [81], [82], the small *ban* hairpin is derived from a ∼40 kb locus, and a 12 kb primary transcript (Figure 4A). Yki, Sd, and Hth binding is extensive across this locus, especially within a large intergenic region 5′ to the start of the primary transcript. Two Gal4 enhancer traps near the promoter of this primary transcript each capture *bantam*'s expression pattern (Figure 4A–C and data not shown). In the eye, expression is high in the proliferative domain anterior to the morphogenetic furrow; in the wing, expression is high in the pouch and hinge, with regions of repression at the dorsal-ventral (DV) and anterior-posterior (AP) compartment boundaries (Figure 4B,C). The fact that both enhancer traps are inserted >12 kb upstream of the hairpin, close to the putative start of transcription, suggests that regulatory inputs driving *ban* expression may be in the large 5′ intergenic region, consistent with the Yki, Sd, and Hth binding patterns.

**Figure 5:**
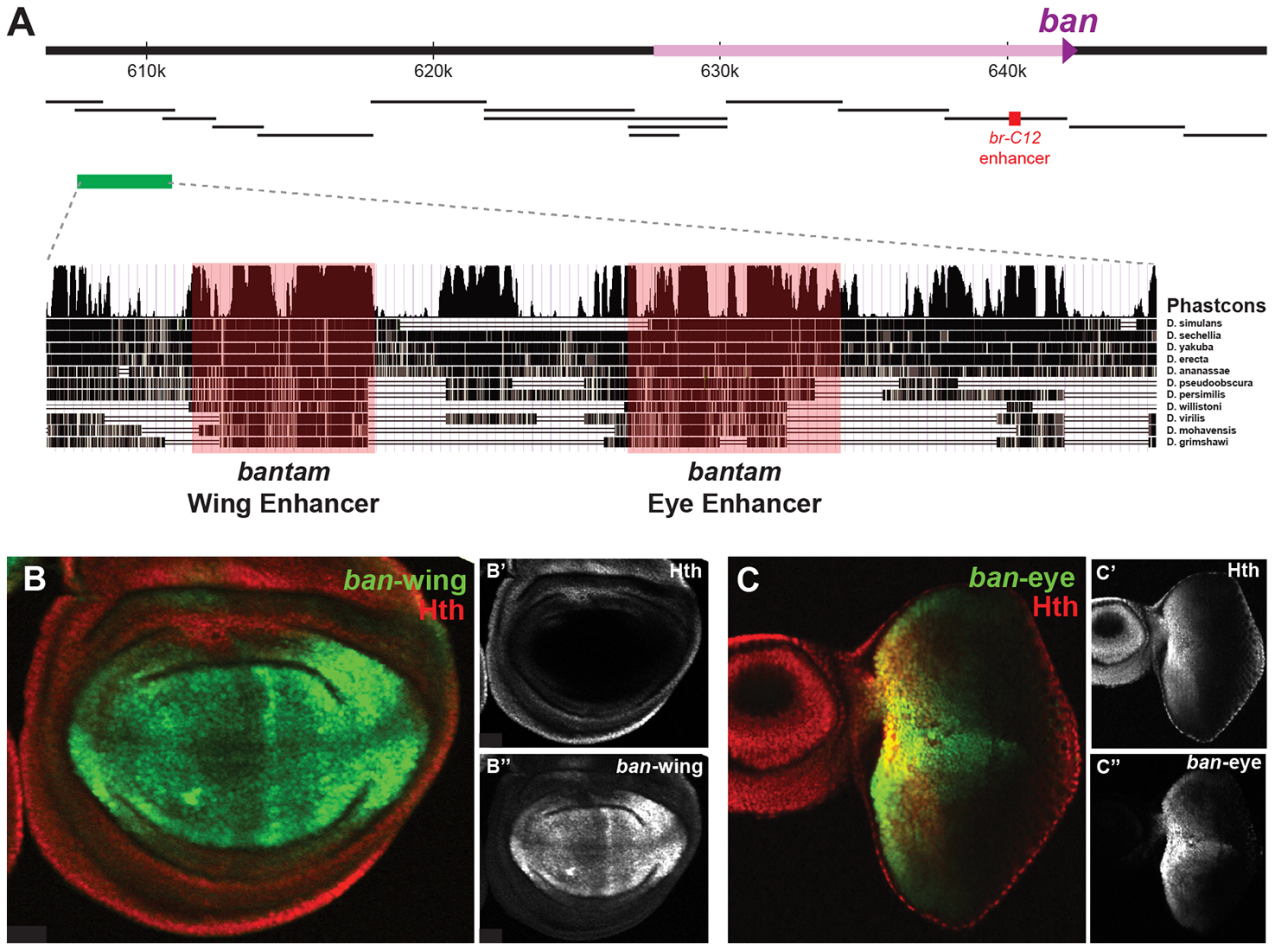
*bantam* eye and wing enhancers. (A) The *bantam* locus is represented. DNA fragments spanning ∼50 kb of the locus were tested for eye and wing expression patterns (black bars). The previously identified Hippo responsive *br-C12* enhancer is marked in red [47]. One region, marked by the green bar, drove expected eye and wing expression patterns. Further dissection of this region identified separate eye and wing enhancers, and PhastCons comparisons highlight their higher degree of conservation. (B) Expression of a *bantam wing-lacZ* transgene (green; see also B″) in a wing imaginal disc. Hth expression is red (see also B′). The *ban-wing* enhancer is not active in the eye (not shown). (C) Expression of a *bantam eye-lacZ* transgene (green; see also C″) in an eye imaginal disc. Hth expression is red (see also C′). The *ban-eye* enhancer is not active in the wing (not shown).

We used transgenic reporter constructs to identify the regulatory modules directing *bantam*'s wing and eye expression, ultimately scanning >40 kb of the *ban* locus (Figure 5A). Although the Gal4 enhancer traps described above are inserted in opposite directions flanking a region of Yki, Sd, and Hth binding, the DNA fragment separating these enhancer traps does not drive *ban*-like expression patterns (not shown). Further searches identified a 3.5 kb region >30 kb upstream of the *ban* hairpin (∼17.5 kb upstream of the putative transcription start site), bound by Sd, Hth, and Yki in both the eye-antenna and wing discs, that recapitulates *ban* eye and wing expression (Figure 5A). None of the other regions tested, including fragments that show strong binding and one that is activated when Hippo signaling is compromised [47], drove a *bantam*-like expression pattern in wing or eye discs. The 3.5 kb region that drives expression in the eye and wing discs was further broken down into distinct eye and wing enhancers (Figures 5B,C). The minimized eye and wing enhancers are 670 bp and 591 bp, respectively, and both are highly conserved across all 12 sequenced *Drosophila* species (Figure 5A).

**Figure 6:**
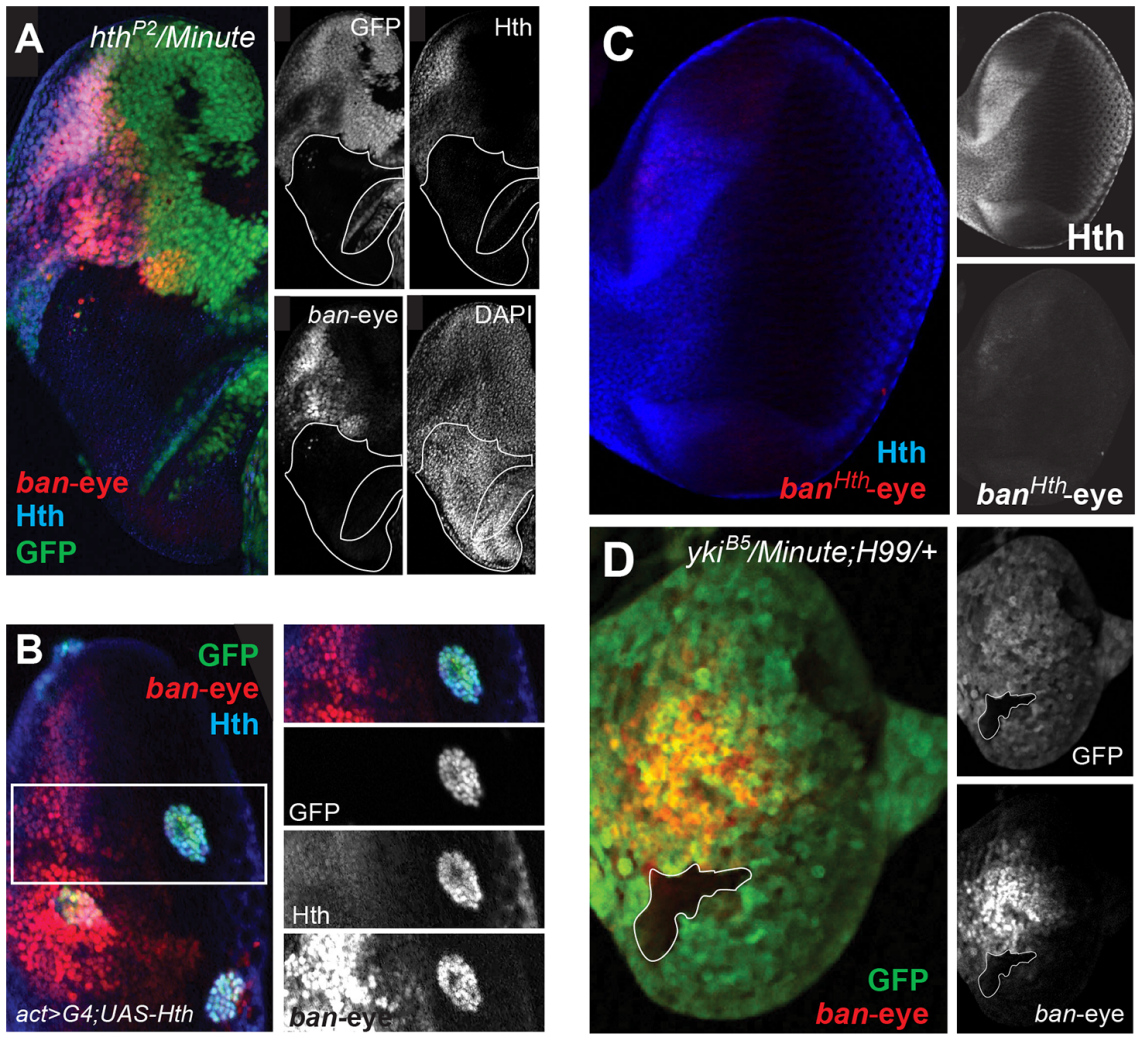
Hth and Yki regulate the *bantam* eye enhancer. (A) *ban-eye-lacZ* expression is lost in *hth^P2^* clones (genotype: *hth^P2^ Minute+*). Clones are marked by absence of GFP; Hth staining is blue and *ban-eye-lacZ* staining is red. Grayscale versions of GFP, Hth and *lacZ* are shown in the panels on the right; DAPI staining was included to show that the tissue remained intact in the *hth^P2^* clone. (B) *ban-eye-lacZ* is upregulated in clones overexpressing Hth (genotype: *act>Gal4; UAS-hth; UAS-GFP*). GFP marks flip-out clones expressing Hth driven by the actin promoter; Hth staining is blue, and *ban-eye-lacZ* staining is red. Grayscale versions of GFP, Hth and *ban-eye-lacZ* are to the right. (C) *ban-eye-lacZ* with the single Hth motif mutated (*ban^Hth^-eye-lacZ*) is not expressed. LacZ staining is in red and Hth is in blue, with grayscale images in panels on the right. (D) *ban-eye-lacZ* expression is lost in a *yki^B5^* clone (genotype: *yki^B5^ Minute+; H99/+*); the clone is marked by the absence of GFP, LacZ staining is in red, and grayscale images are on the right.

Expression of *ban* in the anterior eye progenitor domain is dependent on Hth [48]. Based on ChIP-PCR studies, we previously suggested that Hth and Yki directly activate *ban* in the anterior eye [48]. The identification of the eye enhancer (*ban-eye*) allowed us to further test this hypothesis. The ChIP-chip data indicate that Hth binds this enhancer *in vivo*. Although Yki is also present, it falls below a FDR of 25% (Figure 4A). Nevertheless, the importance of these interactions is demonstrated by additional genetic experiments. Expression driven by the *ban-eye* enhancer is lost in *hth^P2^* clones (Figure 6A) but is unaffected in *sd^ΔB^* clones (not shown). Similar loss of expression was also seen using a *hth* allele (*hth^100.1^*) that only expresses homeodomain-less isoforms of Homothorax, suggesting that full-length Hth is required for activation of *bantam* in the anterior eye (Figure S4A). Expression is also lost in clones of cells lacking *extradenticle* (*exd*), an obligate Hth binding partner (Figure S4B) [83]–[85]. Additionally, the enhancer is strongly activated in clones ectopically expressing Hth posterior to the morphogenetic furrow, in regions of the disc where neither *hth* nor *bantam* are normally expressed (Figure 6B). Importantly, the *ban-eye* enhancer contains a single sequence that matches a Hth binding site, and a 3 bp mutation of this motif (from GACAG to GGGGG) abolished its activity (Figure 6C). The *ban-eye* enhancer also contains a DNA binding motif for Exd, and mutation of this motif (from TGAT to GGGG) resulted in a similar ablation of expression in the eye imaginal disc (Figure S4C). Finally, *ban-eye* expression is also dependent on *yki*, as it was lost in *yki^B5^* clones (Figure 6D). Together with the ChIP data, these genetic and enhancer mutagenesis experiments support a model in which Hth+Exd+Yki directly activate *bantam* expression in the progenitor domain of the eye via an enhancer more than 30 kb upstream of the *bantam* hairpin.

**Figure 7:**
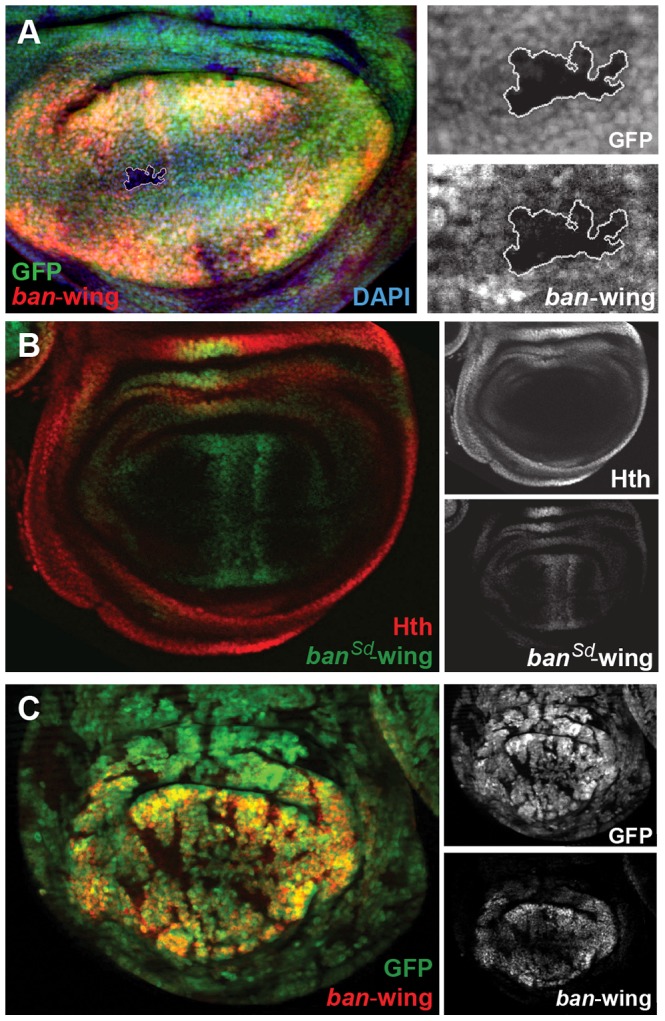
Sd and Yki regulate the *bantam* wing enhancer. (A) *ban-wing-lacZ* expression in lost in a *sd^ΔB^* clone (marked by absence of GFP and outlined in white; genotype: *sd^ΔB^ Minute+*), LacZ staining is in red. Expanded grayscale images of the sdΔB clone (GFP, *ban*-wing) are in panels on right. (B) *ban-wing-lacZ* with seven Sd motifs mutated (*ban^Sd^*-wing). *ban^Sd^-wing-lacZ* staining is in green, and Hth is in red, with grayscale images in panels on the right. Although most *lacZ* expression is gone, weak expression remain on either side of the AP compartment boundary. (C) *ban-wing-lacZ* expression is lost in *yki^B5^* clones (marked by the absence of GFP; genotype: *yki^B5^ Minute+; H99/+*); LacZ staining is in red, and grayscale images are on right.

We carried out similar experiments on the newly identified *bantam* wing enhancer (*ban-wing*). Sd and Yki are required for expression of *bantam* in the wing imaginal disc [49]. Expression driven by the *ban-wing* enhancer is lost in *sd^ΔB^* clones (Figure 7A). To test whether Sd regulation of this enhancer is direct, putative Sd binding sites in the wing enhancer were mutated. Altogether, the *bantam* wing enhancer contains seven putative Sd binding sites, and mutation of all seven eliminated the vast majority of expression in the wing pouch and wing hinge (Figure 7B). Mutation of fewer than seven of the Sd motifs led to more subtle decreases in expression (not shown). Despite the significant loss of wing expression when Sd sites are mutated, residual expression remains in cells flanking the AP compartment boundary in the wing pouch, suggesting that this enhancer may also integrate Decapentaplegic (Dpp) input independently of Sd. These observations are consistent with a previous report showing that Dpp is an activator of *bantam* expression in a Yki-dependent manner [47]. In addition, similar to the *ban-eye* enhancer, expression driven by the *ban-wing* enhancer is lost in *yki^B5^* clones, in all regions of the wing pouch (Figure 7C). Finally, unlike *ban-eye*, mutating the only recognizable Hth binding site had no effect on the activity of the *ban-wing* enhancer (not shown). Together these results suggest that Sd+Yki directly regulate the *bantam* wing enhancer and that Dpp+Yki independently regulate this element close to the AP compartment boundary.

### Regulatory specificity and chromatin environment

**Figure 8:**
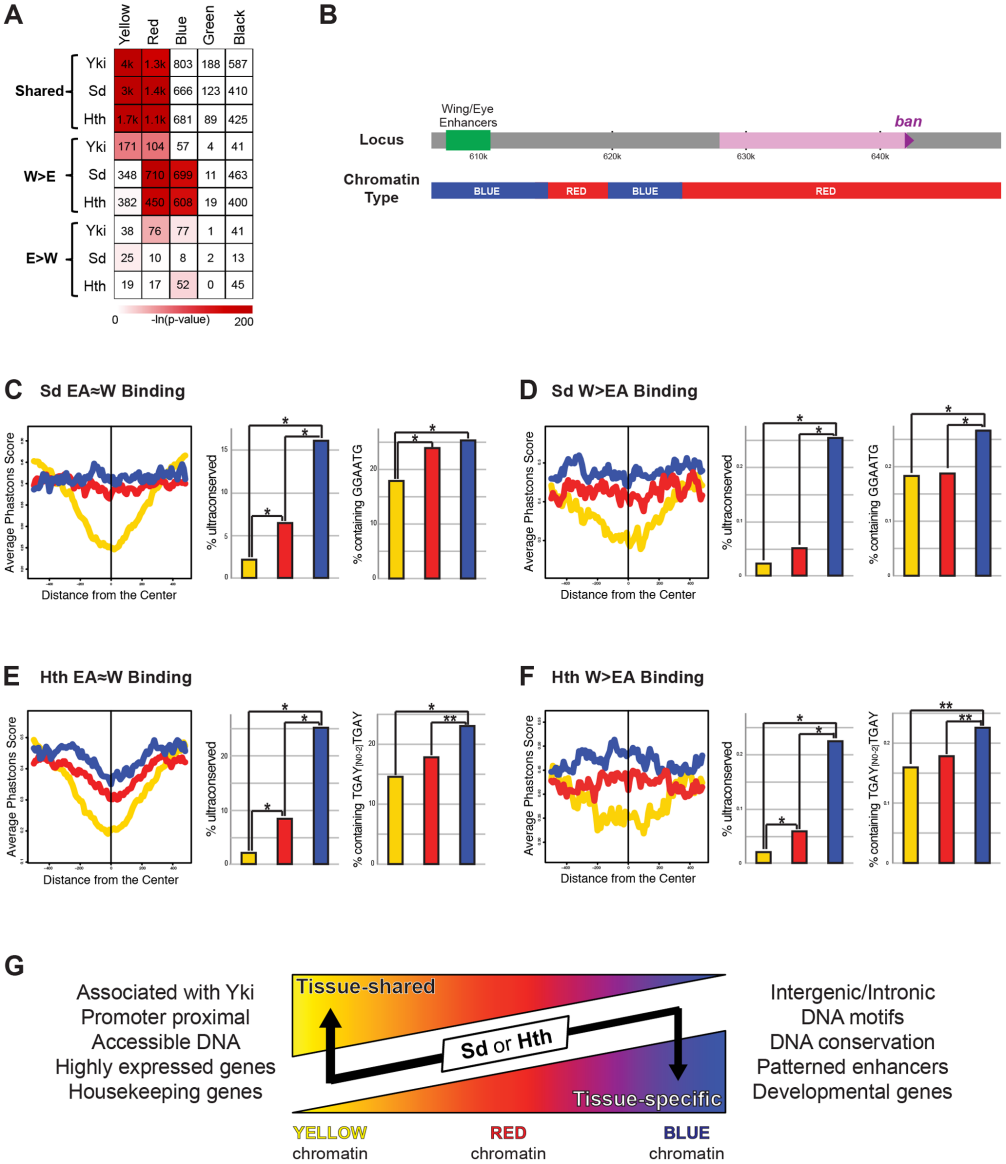
Relationship between Yki, Sd, and Hth binding and chromatin state. (A) A heatmap representing the fraction of EA≈W, W>EA, and EA>W Yki, Sd, and Hth binding regions that overlap the five chromatin states (YELLOW, RED, BLUE, GREEN, and BLACK). Shading represents −ln(p-value) for each pair-wise comparison, and numbers represent the actual number of binding events overlapping a given chromatin state. (B) Schematic representation of the chromatin states across the *bantam* locus. (C–D) Patterns of conservation and motif enrichment at EA≈W and W>EA Sd binding regions. Right panels: average phastcons score +/−500 bp surrounding the peak for (C) EA≈W Sd peaks and (D) W>EA Sd peaks falling in BLUE, RED, or YELLOW chromatin. Middle panels: Percent of peaks that overlap an ultraconserved element as defined in [109], see also Figure 2E. Right panels: Percent of binding regions containing Sd target motif (GGAATG) for (C) EA≈W Sd peaks or (D) W>EA Sd peaks falling in BLUE, RED, or YELLOW chromatin. Lines and bars within graphs are color-coded according to chromatin type. (E–F) Patterns of conservation and motif enrichment at EA≈W and W>EA Hth binding regions. Right panels: average phastcons score +/−500 bp surrounding the peak for (E) EA≈W Hth peaks and (F) W>EA Hth peaks falling in BLUE, RED, or YELLOW chromatin. Middle panels: Percent of peaks that overlap an ultraconserved element as defined in [109], see also Figure 2E. Right panels: Percent of binding regions containing Hth-Exd target motif (TGAY[N0-2]TGAY) for (E) EA≈W Hth peaks or (F) W>EA Hth peaks falling in BLUE, RED, or YELLOW chromatin. Lines and bars within graphs are color-coded according to chromatin type. (G) Summary of properties for tissue-shared and tissue-specific binding by Sd and Hth. Although exceptions exist, in general, binding events for Sd and Hth fall in regions of YELLOW, RED, or BLUE chromatin. Both types of binding are significantly localized to RED chromatin; tissue-shared binding is uniquely biased toward YELLOW chromatin; tissue-specific binding is uniquely biased toward BLUE chromatin. Tissue-shared binding tends to be associated with Yki, promoter proximal, associated with accessible DNA, and highly expressed housekeeping genes. Tissue-specific binding also tends to be distal to the promoter, and is associated with conserved DNA, the expected DNA binding site motifs, enhancers driving patterned expression, and developmental genes.

Genome-wide TF-DNA interactions take place in the context of chromatin, which has the potential to significantly impact a TF's ability to bind DNA [1], [17], [86]. An analysis of dozens of chromatin-associated proteins and histone modifications in *Drosophila* Kc cells generated a high-resolution view of various chromatin states across the genome [34]. Five states were defined that included highly ‘active’ regions (the YELLOW and RED chromatin states), a Polycomb bound region (the BLUE state), and two transcriptionally silent regions (the BLACK and GREEN states). To determine if there is a correlation between chromatin state and Yki, Sd, and Hth binding we looked at the significance of overlap between the binding of these factors and the five chromatin states. Although the small number of EA>W binding events prevented us from finding any significant patterns in the eye-antenna, some interesting patterns emerged when comparing EA≈W and W>EA binding patterns (Figure 8A). All EA≈W binding events are highly enriched for binding in the YELLOW and RED chromatin types (64%, 58%, and 44% of EA≈W Yki, Sd, and Hth sites, respectively, overlap YELLOW chromatin; and 20%, 27%, and 30% of EA≈W Yki, Sd, and Hth sites, respectively, overlap RED chromatin.) Although distinct, both RED and YELLOW are transcriptionally active chromatin states in Kc cells. YELLOW and RED chromatin types are also enriched for Yki W>EA binding events, albeit to a lesser degree than EA≈W, and a significant overlap with RED chromatin is seen for Sd and Hth W>EA peaks. Interestingly, however, for both Sd and Hth, W>EA binding events are not enriched for the YELLOW chromatin state but are instead enriched for binding in BLUE chromatin regions (discussed below): 32% and 30% of Sd and Hth W>EA binding events, respectively, occur in BLUE chromatin. Thus, the tissue-specific and tissue-shared binding for Sd and Hth correlate with distinct chromatin landscapes.

The correlations between TF binding and distinct chromatin types become more interesting when considering the properties of YELLOW, RED, and BLUE chromatin. YELLOW chromatin, which is preferred by all three factors but only at sites with tissue-shared binding, is associated with active chromatin modifications and genes that are highly expressed in a ubiquitous manner (ribosomal components, DNA repair machinery, etc.) [34]. The DNA in RED chromatin, which is enriched in both tissue shared and W>EA binding by Sd, Hth, and Yki, is highly accessible, as measured by FAIRE (formaldehyde assisted isolation of regulatory elements) [87], and associated with genes expressed in a patterned fashion, such as genes involved in signal transduction and those encoding transcription factors [34]. BLUE chromatin, where only W>EA Sd and Hth binding events are highly enriched, is marked by Polycomb group (PcG) proteins and PcG-associated repressive histone modifications (histone H3 lysine 27 trimethylation); genes associated with BLUE chromatin tend to encode exquisitely controlled developmental master regulator genes (i.e., selector and selector-like genes). The above patterns are also apparent when looking at enriched Gene Ontology (GO) categories (Dataset S6). In addition, GO analysis reveals that the small number of EA>W binding events for Hth and Sd are also associated with selector-like genes: retinal determination and photoreceptor specification genes, respectively (Dataset S6). Thus, for Sd, Hth, and Yki, EA≈W binding is strongly associated with genes that are highly and ubiquitously expressed. Tissue-specific Sd and Hth binding, on the other hand, is uniquely enriched in highly regulated selector-like gene loci (see Dataset S6 for examples).

Although tissue-specific binding is abundant for both Sd and Hth, the *bantam* wing and eye enhancers direct tissue-specific, patterned expression even though overall Sd/Hth/Yki binding is similar between both tissues at these regions (Figure 4). These enhancers may provide an example of how context-independent input from Sd and Hth can drive developmentally patterned expression. Alternatively, because our ChIP experiments were carried out with whole wing and whole eye-antenna discs, it is also possible that the observed ChIP signals come from distinct cell types within individual discs (e.g. Hth may bind to the *ban-wing* enhancer in the hinge and Sd may bind to the *ban-wing* enhancer in the pouch). Consistent with this notion, these CRMs are in BLUE chromatin, where tissue-specific binding is typically observed (Figure 8). In fact, although Sd and Hth tissue-shared binding is most enriched at YELLOW chromatin, ∼10–20% of these binding events occur in BLUE chromatin and ∼30% occur in RED chromatin.

Finally, we explored the properties of tissue-shared Sd- or Hth-bound DNA across different chromatin types. First, the DNA bound by these factors in BLUE and RED chromatin is much more conserved than that falling within YELLOW chromatin (Figure 8C and 8E). Second, when we search for the DNA motifs identified in the W>EA binding events (Figure 2) we find that these motifs are significantly more enriched at peaks in BLUE and RED chromatin compared to YELLOW chromatin (Figure 8C and 8E). For Sd, the core GGAATG sequence is significantly more enriched in tissue-independent binding events occurring in BLUE and RED chromatin (Figure 8C). Similar patterns are seen with the more degenerate sequence RGAATG, with >39% of BLUE peaks containing the motif and <35% of YELLOW peaks containing the motif (p = 0.0257). For Hth, neither of the identified consensus sequences (TGAC or TGAT) is differentially enriched in the various chromatin states (not shown). However, as mentioned above, Hth binds DNA together with a second homeodomain-containing factor, Exd. The core overrepresented sequences from Figure 2, TGAC and TGAT, represent Hth and Exd consensus motifs, respectively, and adjacent copies of these motifs are significantly enriched in BLUE chromatin binding events (Figure 8E). A similar pattern is observed for the more degenerate sequence (WGAY{N_0–2_}WGAY), with >51% of BLUE peaks and >37% of YELLOW peaks containing this sequence (p<0.0001). Interestingly, the same patterns are seen when comparing W>EA binding across BLUE, RED, and YELLOW chromatin (Figure 8D,F), indicating that many of the overall differences between tissue-specific and tissue-shared binding are based on the properties of Sd and Hth binding in YELLOW versus BLUE and RED chromatin. These findings suggest that a subset of tissue-nonspecific binding events, particularly those falling within regions of BLUE chromatin, is likely to regulate developmentally patterned gene expression.

## Discussion

The control of gene expression in multicellular eukaryotes depends on a limited set of transcription factors that are reused in different contexts and combinations to execute a diverse array of cellular functions. To gain insight into this process we used tissue-specific, genome-wide ChIP to explore the global DNA targeting properties of three transcriptional regulators – Yki, Sd, and Hth. Yki is a transcriptional coactivator that regulates tissue growth in all tissues, and it does so in part through interactions with the DNA binding TFs Sd and Hth [41], [48]–[51]. However, in addition to their Yki-dependent roles in promoting tissue growth, Sd and Hth also have highly tissue-specific developmental roles [52]–[59]. Thus, this group of regulators provides an ideal starting point for addressing the logic by which TFs execute both tissue-specific and -nonspecific gene regulatory functions in vivo. Below we discuss the implications of the differences we uncovered between these modes of binding for Hth and Sd, as well as the unexpectedly large number of shared binding sites for Yki.

*Drosophila* Yki was initially identified as an essential transcriptional coactivator in the Hippo tumor suppressor pathway [45]. Loss of function clones of *yki* grow very poorly, while gain of function Yki clones result in tissue overgrowths that are similar to those generated when the upstream kinases (Hippo and Warts) are compromised. These observations suggested that Yki, with the help of DNA binding proteins, would target genes required for cell proliferation and survival, including the known Hippo pathway targets *cycE* and *diap1*. Consistent with this expectation, we observe Yki binding to these and other genes that are regulated by the Hippo pathway (Figure S3). Unexpectedly, however, in addition to known Hippo pathway genes we observe Yki binding to several thousands of genes in both the eye-antenna and wing imaginal discs, implying that Yki targets many more genes than those regulated by the Hippo pathway, or that the Hippo pathway targets many more genes than previously thought. Consistent with the latter possibility, over 1000 of the genes identified as tissue-shared Yki targets in this study are upregulated >2-fold in *wts* ^−^ wing discs relative to wild-type based on recently published RNA-seq data [88]. In addition, Yki was recently shown to bind and activate several genes required for mitochondrial fusion [89]. Moreover, the mammalian homologs of Yki, Yes-associated protein (YAP) and TAZ (transcriptional coactivator with PDZ-binding motif) are thought to regulate many genes in a wide variety of contexts, including human embryonic stem cells and several adult human tissues [39], [41], [42]. Taken together, these results suggest that Yki may be a widely used transcriptional coactivator in Drosophila and vertebrates. The severe cell proliferation defects associated with *yki* mutant clones may have obscured its other functions in other pathways. These results are consistent with the idea that Yki and its vertebrate orthologs interact with a wide variety of transcription factors [47], [88], [90], [91]. Together, the data imply that DNA binding proteins in addition to Sd and Hth may recruit Yki to a large number of broadly active CRMs.

The view that Yki is recruited to DNA by factors other than Sd was recently questioned by experiments suggesting that, in the eye imaginal disc, *sd yki* double mutant clones proliferate better than *yki* single mutant clones [92]. These observations were interpreted to suggest that Sd is a default repressor of proliferation and survival-promoting genes. However, this conclusion is complicated by the observation that both Sd and Yki are also important for specifying non-retinal (peripodial epithelium) fates in the eye imaginal disc [60]: thus, the partially rescued growth of *sd yki* clones could in part be due to a fate transformation. Further, we found that the activity of the *ban-eye* enhancer is not affected in *sd* clones, but is lost in *hth* clones, arguing that at least for this direct Hippo pathway target Hth, not Sd, is the primary activator. It is noteworthy that although their activities can be separated, the *ban* wing and eye enhancers identified here are adjacent to each other in the native *ban* locus. It is plausible that Sd+Yki input provides a basal level of activity in both tissues and that Hth and Sd boost this level in the eye and wing, respectively. Regardless, the improved growth of *sd yki* clones does not argue against the idea that Yki is recruited to survival genes by Hth in wild type eye discs. Taken together with our genome-wide binding and *ban* enhancer studies, we suggest that the absence of Sd results in both a fate change and some derepression of survival genes, but that wild type proliferation and gene regulation in the eye disc requires the recruitment of Yki to the DNA by Hth.

In contrast to the widespread and largely tissue-nonspecific binding we observe for Yki, Sd and Hth exhibit both tissue-specific and tissue-shared binding events. Multiple characteristics distinguish these types of binding. First, tissue-shared binding by both Sd and Hth is frequently associated with Yki binding and often close to cell cycle and housekeeping genes, while tissue-specific binding is not. These observations are consistent with previous studies showing that Yki controls cell survival and proliferation in all imaginal discs, an activity that is regulated by the Hippo pathway [45], [62]. Second, compared to tissue-shared binding, DNA sequences bound by Sd and Hth in a tissue-specific manner are more conserved, more likely to contain the TF's consensus binding site, less likely to be promoter proximal, and more likely to be associated with key developmental regulatory loci. Third, tissue-specific Sd and Hth binding events are more likely to overlap with enhancers active in the corresponding tissue. To illustrate this point, the newly identified tissue-specific TF-CRM interactions at *wg* match the known roles for Sd and Hth (Figure 3B). Taken together, these results suggest that regulation at the level of TF-DNA binding is a significant mechanism by which Sd and Hth regulate tissue-specific gene expression. Tissue-specific binding could be regulated through direct or indirect interactions with additional transcription factors, through tissue-specific differences in DNA accessibility, or through a combination of these factors.

We also found that distinct chromatin types are differentially correlated with tissue-specific and -nonspecific binding, even though these chromatin categories were defined in Kc cells. All tissue-shared binding events have a strong tendency to occur in actively transcribed chromatin states (YELLOW and RED). Tissue-specific (W>EA) Sd and Hth binding is also enriched in RED chromatin but is uniquely enriched in BLUE chromatin. BLUE chromatin is associated with Polycomb-mediated repression. The W>EA Sd and Hth binding in Polycomb-associated chromatin indicate that these factors target tissue-specific enhancers that are also regulated by PcG proteins during development.

Despite the importance of tissue-specific binding as a regulatory mechanism for Sd and Hth activity, both factors also displayed a significant amount of tissue-shared binding. We found that these tissue-shared binding events can be broken down into distinct groups based on the local chromatin environment (Figure 8G). The majority of tissue-shared binding occurs in YELLOW chromatin and is associated with ubiquitously expressed housekeeping genes. However, binding that occurs in BLUE chromatin, and to a lesser extent in RED chromatin, is more conserved and more likely to be associated with a TF's motif, both characteristics of tissue-specific binding. In the case of the *bantam* eye and wing enhancers, Sd and Hth binding in BLUE chromatin is direct and apparently able to drive tissue-specific, rather than ubiquitous, expression patterns. Other examples of enhancers in RED or BLUE chromatin that drive patterned expression and have tissue shared binding are shown in Figure S5. These observations suggest that gene regulation by Sd and Hth may also be controlled at a step beyond DNA binding, perhaps via interactions with additional transcription factors at a given enhancer. Alternatively, some of the binding events called as tissue-shared may turn out to be specific binding events in distinct cell types within each imaginal disc (e.g. hinge, notum, and pouch in the wing disc and antenna, eye progenitor domain, and photoreceptors in the eye-antenna disc). Regardless, the hundreds of Sd- and Hth-CRM interactions identified in this study (Dataset S5) provide a tremendous resource for further dissecting the mechanisms by which Sd and Hth regulate patterned gene expression.

Notably, few of the above conclusions would have been clear had genome-wide binding been measured in only one of the two tissues. Tissue-specific binding is not the most highly enriched (that is, the signal is generally weaker compared to tissue-shared events) (Figure S6) and might have been overlooked had we just characterized one tissue, where the strongest peaks are generally the focal point [7], [10], [93]. The tissue-specific binding events detected here may also occur in subsets of cells in the wing or eye-antennal discs, which are also heterogeneous in cell type. This would explain why tissue-specific binding signals may be weaker, because the ChIP data represent an average of all cell types in a single imaginal disc type. If correct, it would be an error to focus on only the strongest peaks when analyzing *in vivo* TF binding, particularly in heterogeneous tissues. It is possible that ChIP signal is more biologically meaningful in highly homogenous tissues like the blastoderm *Drosophila* embryo, or in cell culture. Still, distinct TF-DNA binding mechanisms (long residence time versus rapid binding turnover) with different functional outcomes can lead to indistinguishable, strong ChIP peaks, making it difficult to interpret ChIP data on strength of signal alone [94]. Despite their lower intensity, many biologically relevant binding events, such as those identified here, may only stand out when looking at the influence of tissue context on binding.

## Materials and Methods

### Chromatin immunoprecipitation and ChIP-chip

Imaginal disc ChIPs were performed as described previously [63], [95]. Briefly, imaginal discs were dissected from wandering third-instar larvae and placed in PBS on ice. Discs were then fixed with 1.8% formaldehyde, and chromatin was sonicated to an average size of 500 bp. Immunoprecipitations were performed with goat anti-Hth (dG-20, Santa Cruz Biotechnologies; 1.5 µg/ml for IP), rabbit anti-GFP (ab290, Abcam; 1∶300 dilution for IP), and rabbit anti-Yki ([62]; 1∶300 dilution for IP). ChIP and input DNA were amplified using the GenomePlex WGA4 Whole Genome Amplification Kit (Sigma), and then labeled according to Affymetrix protocols and hybridized on Affymetrix GeneChip Drosophila Tiling 2.0R Arrays.

### ChIP-chip data analysis

Tiling array data were processed with MAT (Model-based Analysis of Tiling-arrays), with peaks called at 1% FDR (false discovery rate) and 25% FDR as described in the results section [64]. FlyLight enhancers were described previously [72], [96]. The significance of overlap between genomic regions (ChIP peaks versus ChIP peaks; ChIP peaks versus FlyLight enhancers; ChIP peaks versus chromatin states) were calculated using the mergePeaks program within the HOMER (Hypergeometric Optimization of Motif EnRichment) Suite [97]; expected overlap and co-occurrence p-values are calculated based on the hypergeometric distribution. Breakdown of binding events by genomic region was performed using the CEAS (Cis-regulatory Element Annotation System) program within the Cistrome platform [98]. Motif analysis in Figure 2 was performed using Centrimo, which uses a binomial test to identify non-randomly distributed motifs within ChIP peaks (i.e., motifs selectively enriched near peak centers) [99]. The JASPAR Core database [100] was used for motif scanning by Centrimo, with the default significance threshold of *E*-value ≤10; *E*-value is the enrichment *p*-value (binomial test) multiplied by the number of motifs in the JASPAR Core database (460 motifs).

### *Drosophila* genetics

Two Gal4 enhancer trap insertion lines, coupled with *UAS-GFP*, were used to assess *bantam* expression pattern: NP3256 and NP0016 (DGRC, Kyoto). Ectopic Hth expression was examined on larvae with genotype: *yw,hs-Flp^1.22^; ban-eye(51D)/UAS-Hth; actin>stop>Gal4, UAS-GFP/+*; larvae were heat-shocked for 7 min at 37°C and dissected 48 h later at crawling stage (*UAS-Hth* is described in [101]). All mutant clones were performed on *Minute* background and in some cases in *Df(3L)H99* (*hid*-, *rpr*-, *grim*-)/+ background [102] in order to alleviate the growth disadvantage of these mutant cells. *hth* mutant clones were analyzed in imaginal discs from non-*Tb* larvae that resulted from the cross between males with genotype *yw;; FRT82B hth^P2^/TM6B,Tb* or *yw;; FRT82B hth^100-1^/TM6B, Tb* and females with genotype *yw, hs-Flp^1.22^; ban-eye(51D); FRT82B, M, hs-GFP/TM6B, Tb*. *exd* mutant clones were analyzed in discs from female larvae from the progeny of males with genotype *yw, exd^1^, FRT19A/Y; tub-Exd/CyO* (the *tub-Exd* transgene rescues the mutant *exd^1^* [103]) and females with genotype *yw, M, Ubi-GFP, FRT19A/FM7; ban-eye(51D); hs-Flp*. *exd* mutant clones were verified by lack of staining with anti-Exd Ab. *sd* mutant clones were analyzed in discs from *Tb* female larvae that resulted from the cross between males with genotype *yw, sd^ΔB^, FRT19A/Y;; Dp(1;3)DC523/TM6B, Tb* (*Dp(1;3)DC523* rescues the *sd* mutant) and females with genotype *yw, M, Ubi-GFP, FRT19A/FM7; ban-wing(51D); hs-Flp*. *yki* clones were analyzed in discs from larvae resulting from the cross between males with genotype *yw, hs-Flp^1.22^; FRT42D, yki^B5^; Df(3L)H99/C(2L;3R),Tb* and females with genotype *yw, hs-Flp^1.22^; FRT42D, M, hs-GFP/Cyo; ban-lacZ(86Fa)/TM2*, where *ban-lacZ* is either the *ban-wing* or *ban-eye* enhancer driving *lacZ*. All mitotic clones where generated by a 45 min heat shock at 37°C and because of the *Minute* background larvae were dissected 72 h after heat shock at crawling stage. To induce GFP expression in larvae marked with *hs-GFP*, those were heat-shocked again 1 h before dissection for 20 min at 37°C.

### Vectors and transgenes

All *enhancer-reporter in vivo* assays were performed using the PhiC31 attB/attP system [104]. Overlapping DNA fragments covering >40 kb of the *bantam* locus were PCR amplified and introduced in *lacZ*-bearing reporter vectors. Two attB reporter vectors were used for assaying enhancer activity: one marked with *mini-white+* gene (pRVV54) and the other marked with *mini-yellow+* gene (pRVV212). Both vectors carry a multiple cloning site for enhancer introduction, a minimal *Drosophila* synthetic core promoter [105], followed by nuclear *lacZ* [106] and the late SV40 transcriptional terminator sequence [107]. Transgenes were inserted in either 51D or 86Fa attP sites [108]. Once minimized the *ban* eye and wing enhancers were inserted in both 51D and 86Fa attP sites and used for genetic experiments. Mutant *ban* enhancer transgenes were inserted in site 51D and compared to the corresponding (eye or wing) wildtype *ban* enhancer transgene inserted in the same site. The *bantam* eye enhancer is delimited by primers: GCTTCGCATCGTAGTCGTCCCCC and TAAAAAAAAAAAACAGAAGCACCTTTG. The *bantam* wing enhancer is delimited by primers: GTTTGCTCTGCTCTACGCCACC and AACTTTCAACTTTTTTTTTTAGTTG. Primers used for enhancer mutagenesis are listed in Table S1.

### Immunostaining

The following antibodies were used for tissue stainings: rabbit anti-β-galactosidase (Cappel), guinea pig anti-Hth [85], rabbit anti-Exd (8857540), rabbit anti-Yki (gift from D. Pan), mouse anti-Dlg (Developmental Studies Hybridoma Bank). Imaginal discs were immunostained by standard procedures. AlexaFluor488, AlexaFluor555, and AlexaFluo647 conjugates with secondary antibodies from Invitrogen were used at 1∶1000 dilution.

## Supporting Information

Dataset S1 Yki, Sd, and Hth ChIP peaks and putative target genes.
(XLSX)

Dataset S2 Motifs enriched in Sd and Hth EA≈W and W>EA binding regions.
(XLSX)

Dataset S3 Gene Ontology analysis of Sd and Hth EA≈W targeted genes.
(XLSX)

Dataset S4 Expression level of Sd and Hth EA≈W target genes in the wing disc.
(XLSX)

Dataset S5 Yki, Sd, and Hth overlap with all FlyLight enhancers expressed in the wing, eye, antenna, or leg.
(XLSX)

Dataset S6 Gene Ontology analysis of Sd and Hth W>EA and EA>W targeted genes.
(XLSX)

Figure S1 Yki overlap with Sd and Hth binding. (A) Venn diagram representing overlap for Yki, Sd, and Hth peaks called at a false discovery rate of 1% in the wing disc. (B) Venn diagram representing overlap for Yki, Sd, and Hth peaks called at a false discovery rate of 1% in the eye-antenna disc. (C) Heatmap representing the significance of overlap in binding between tissue-shared (EA≈W) and tissue-specific (W>EA or EA>W) Yki peaks and various categories of Sd and Hth binding as indicated. Shading represents −ln(p-value) for each pair-wise comparison. ‘EA≈W only’ represents tissue-shared peaks at genes without additional W>EA Sd or Hth binding. ‘**EA≈W**+W>EA’ represents tissue-shared peaks at genes with additional W>EA Sd or Hth binding. ‘EA≈W+**W>EA**’ represents wing-specific peaks that target genes with additional EA≈W Sd or Hth binding. ‘W>EA only’ represents wing-specific peaks at genes without additional EA≈W Sd or Hth binding. Note that the EA≈W peaks for both Sd and Hth have a strong tendency to overlap Yki EA≈W peaks.
(TIF)

Figure S2 Top motifs enriched in Sd and Hth peaks. Up to the top 10 enriched motifs for Sd and Hth W>EA and EA≈W peaks. At an *E*-value threshold of ≤10 (see methods), only three motifs were significantly enriched for Sd W>EA, whereas all of the others were enriched for 10 or more, often redundant, motifs. For comparison, consensus PWMs for Hth and Sd are shown underneath.
(TIF)

Figure S3 Sd and Hth binding at Hippo pathway targets. (A) Yki, Sd, and Hth binding profiles in the eye-antenna and wing imaginal discs across the *expanded* locus. (B) Yki, Sd, and Hth binding profiles in the eye-antenna and wing imaginal discs across the *thread/diap1* locus. (C) Yki, Sd, and Hth binding profiles in the eye-antenna and wing imaginal discs across the *cyclin E* locus.
(TIF)

Figure S4 Hth and Exd regulate the *bantam* eye enhancer. (A) *ban-eye-lacZ* expression is lost in *hth^100-1^* clones (genotype: *hth^100-1^ Minute+*). Clones are marked by absence of GFP and LacZ staining is in red. Grayscale images are on the right. (A′) Grayscale version of GFP staining. (A″) Grayscale version of LacZ staining. (B) *ban-eye-lacZ* expression in *exd^1^* clones (gentype: *exd^1^ Minute+*). Clones are marked by absence of GFP and lacZ staining is in red. Grayscale images are on the right. (B′) Grayscale version of GFP staining. (B″) Grayscale version of LacZ staining. (C) *ban-eye-lacZ* with Exd motif mutated (*ban^Exd^-eye-lacZ*) is not expressed. LacZ staining is in red, and Hth is in blue, with grayscale images on the right. (C′) Grayscale version of Hth staining. (C″) Grayscale version of LacZ staining.
(TIF)

Figure S5 Patterned expression driven by fragments with tissue-shared peaks. Examples of FlyLight enhancers targeted by tissue-shared binding; all enhancers overlap BLUE or RED chromatin as indicated and have EA≈W binding for both Sd and Hth. In some cases (R78B08, R48H04, R30A01) the enhancer only drives expression in one of the two discs. In all cases expression is highly patterned within a disc, and similar Sd+Hth inputs can lead to distinct expression patterns (e.g., R46A09 is expressed in anterior eye disc while R34D04 is expressed in the posterior eye disc). In all images enhancer-Gal4>UAS-GFP signal is in green, and DAPI or Hth staining is in blue. Images were taken from the FlyLight database [72], [96] and can be found at <http://flweb.janelia.org/cgi-bin/flew.cgi>.
(TIF)

Figure S6 ChIP signal at tissue-shared and tissue-specific peaks. (A) Average ChIP signal (MAT score) +/−500 bp surrounding peaks for tissue-shared (EA≈W) and wing-specific (W>EA) Sd binding. (B) Average ChIP signal (MAT score) +/−500 bp surrounding peaks for tissue-shared (EA≈W) and wing-specific (W>EA) Hth binding.
(TIF)

Table S1 Primers used for *ban* enhancer mutagenesis. Nucleotides targeted in each round of site-directed mutagenesis are indicated in capital letters. Wild-type enhancers were introduced in pBluescript SK+ and mutagenized by site-directed mutagenesis using the above primers and subsequently transferred to pRVV54 (for *ban* wing enhancer) or pRVV212 (for *ban* eye enhancer) to assay enhancer activity *in vivo*.
(DOCX)
