# Supplementary figures and images for: Divergent Transcriptional Regulatory Logic at the Intersection of Tissue Growth and Developmental Patterning
Source: PLoS Genet. 2013 Sep 5;9(9):e1003753. doi: 10.1371/journal.pgen.1003753 (PMC3764184; doi:10.1371/journal.pgen.1003753)

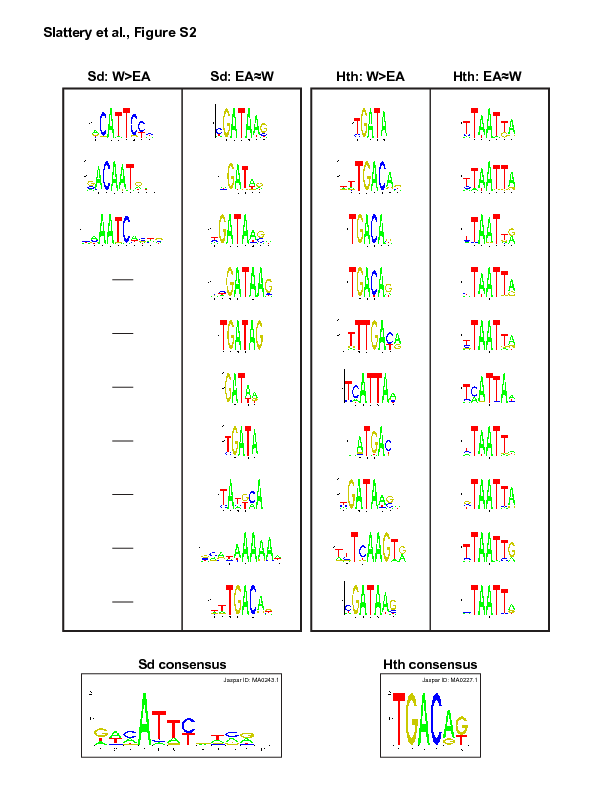

Supplement: Figure S2 — Top motifs enriched in Sd and Hth peaks. Up to the top 10 enriched motifs for Sd and Hth W>EA and EA≈W peaks. At an E-value threshold of ≤10 (see methods), only three motifs were significantly enriched for Sd W>EA, whereas all of the others were enriched for 10 or more, often redundant, motifs. For comparison, consensus PWMs for Hth and Sd are shown underneath. (TIF) [file pgen.1003753.s008.tif]

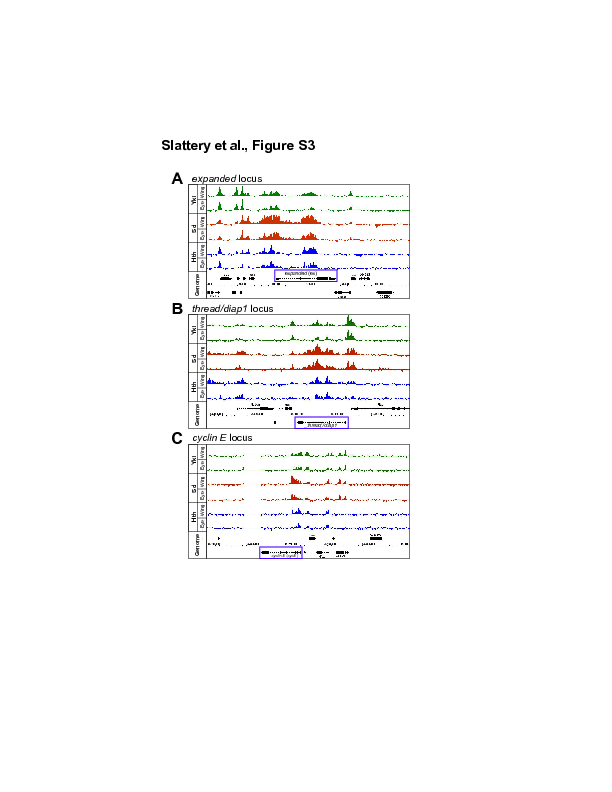

Supplement: Figure S3 — Sd and Hth binding at Hippo pathway targets. (A) Yki, Sd, and Hth binding profiles in the eye-antenna and wing imaginal discs across the expanded locus. (B) Yki, Sd, and Hth binding profiles in the eye-antenna and wing imaginal discs across the thread/diap1 locus. (C) Yki, Sd, and Hth binding profiles in the eye-antenna and wing imaginal discs across the cyclin E locus. (TIF) [file pgen.1003753.s009.tif]

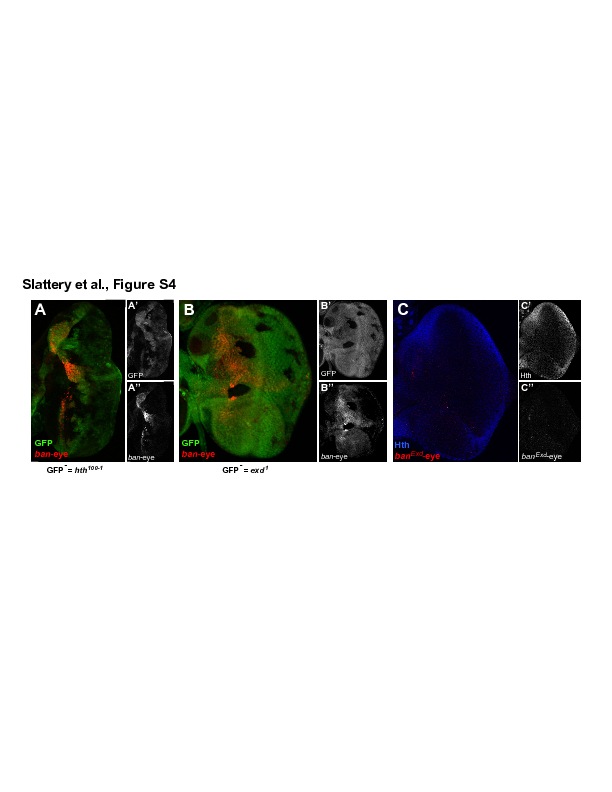

Supplement: Figure S4 — Hth and Exd regulate the bantam eye enhancer. (A) ban-eye-lacZ expression is lost in hth100-1 clones (genotype: hth100-1 Minute+). Clones are marked by absence of GFP and LacZ staining is in red. Grayscale images are on the right. (A′) Grayscale version of GFP staining. (A″) Grayscale version of LacZ staining. (B) ban-eye-lacZ expression in exd1 clones (gentype: exd1 Minute+). Clones are marked by absence of GFP and lacZ staining is in red. Grayscale images are on the right. (B′) Grayscale version of GFP staining. (B″) Grayscale version of LacZ staining. (C) ban-eye-lacZ with Exd motif mutated (banExd-eye-lacZ) is not expressed. LacZ staining is in red, and Hth is in blue, with grayscale images on the right. (C′) Grayscale version of Hth staining. (C″) Grayscale version of LacZ staining. (TIF) [file pgen.1003753.s010.tif]

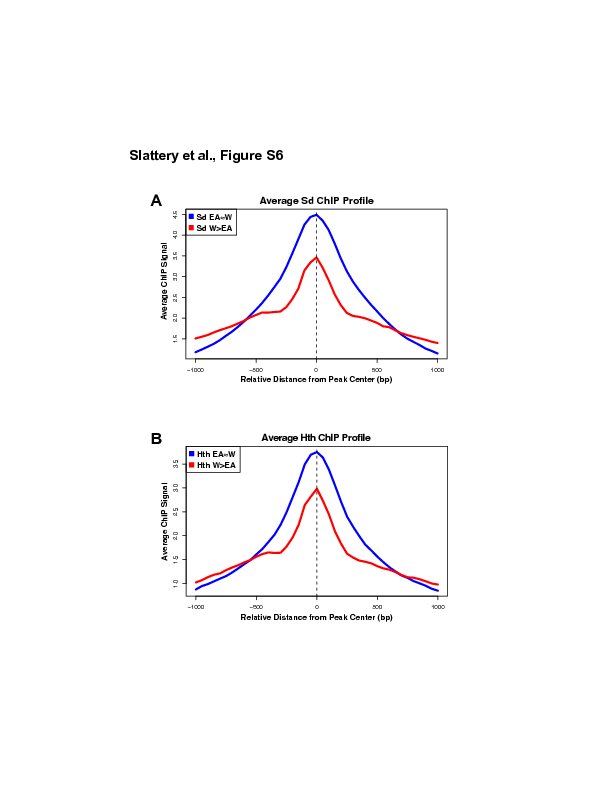

Supplement: Figure S6 — ChIP signal at tissue-shared and tissue-specific peaks. (A) Average ChIP signal (MAT score) +/−500 bp surrounding peaks for tissue-shared (EA≈W) and wing-specific (W>EA) Sd binding. (B) Average ChIP signal (MAT score) +/−500 bp surrounding peaks for tissue-shared (EA≈W) and wing-specific (W>EA) Hth binding. (TIF) [file pgen.1003753.s012.tif]
