# Supplementary material for: Divergent Transcriptional Regulatory Logic at the Intersection of Tissue Growth and Developmental Patterning
Source: PLoS Genet. 2013 Sep 5;9(9):e1003753. doi: 10.1371/journal.pgen.1003753 (PMC3764184; doi:10.1371/journal.pgen.1003753)
Supplement: Table S1 — Primers used for ban enhancer mutagenesis. Nucleotides targeted in each round of site-directed mutagenesis are indicated in capital letters. Wild-type enhancers were introduced in pBluescript SK+ and mutagenized by site-directed mutagenesis using the above primers and subsequently transferred to pRVV54 (for ban wing enhancer) or pRVV212 (for ban eye enhancer) to assay enhancer activity in vivo. (DOCX) [file pgen.1003753.s013.docx]

| **Mutant site** | **Primer sequence** |
| --- | --- |
| Eye Enhancer Hth | gtacgggtttgccgtgataaaggatggGGGggtgattatgacttttttagcggg |
| Eye Enhancer Hth | cccgctaaaaaagtcataatcaccCCCccatcctttatcacggcaaacccgtac |
| Eye Enhancer Exd | ccaaagttgtctgtacgggtttgccgGgGGaaaggatggacaggtgattatg |
| Eye Enhancer Exd | cataatcacctgtccatcctttCCcCcggcaaacccgtacagacaactttgg |
| Wing Enhancer Sd 1 | gagtcaacgactcccctgCCCCttcgtgttggcccttttcg |
| Wing Enhancer Sd 1 | cgaaaagggccaacacgaaGGGGcaggggagtcgttgactc |
| Wing Enhancer Sd 2-3 | gatgccccctttggataccccgTtcTccgTtcTcgggtgcctcgatcctcg |
| Wing Enhancer Sd 2-3 | cgaggatcgaggcacccgAgaAcggAgaAcggggtatccaaagggggcatc |
| Wing Enhancer Sd 4-5 | cctcctccgcccccagTtgTcccctttggaAaAcccgttctccgttctc |
| Wing Enhancer Sd 4-5 | gagaacggagaacgggTtTtccaaaggggAcaActgggggcggaggagg |
| Wing Enhancer Sd 6 | gattggtcgttttaacctgtttgcTaaAttctaccatgttaataaataatt |
| Wing Enhancer Sd 6 | aattatttattaacatggtagaaTttAgcaaacaggttaaaacgaccaatc |
| Wing Enhancer Sd 7 | cgttctccgttctcgggtgcctcgaAcAtcgtgcagctccggctggctgcc |
| Wing Enhancer Sd 7 | ggcagccagccggagctgcacgaTgTtcgaggcacccgagaacggagaacg |

**Slattery et al., Table S1**
